# Tick salivary compounds: their role in modulation of host defences and pathogen transmission

**DOI:** 10.3389/fcimb.2013.00043

**Published:** 2013-08-20

**Authors:** Mária Kazimírová, Iveta Štibrániová

**Affiliations:** ^1^Institute of Zoology, Slovak Academy of SciencesBratislava, Slovakia; ^2^Institute of Virology, Slovak Academy of SciencesBratislava, Slovakia

**Keywords:** ticks, saliva, immunomodulation, pathogen, transmission

## Abstract

Ticks require blood meal to complete development and reproduction. Multifunctional tick salivary glands play a pivotal role in tick feeding and transmission of pathogens. Tick salivary molecules injected into the host modulate host defence responses to the benefit of the feeding ticks. To colonize tick organs, tick-borne microorganisms must overcome several barriers, i.e., tick gut membrane, tick immunity, and moulting. Tick-borne pathogens co-evolved with their vectors and hosts and developed molecular adaptations to avoid adverse effects of tick and host defences. Large gaps exist in the knowledge of survival strategies of tick-borne microorganisms and on the molecular mechanisms of tick-host-pathogen interactions. Prior to transmission to a host, the microorganisms penetrate and multiply in tick salivary glands. As soon as the tick is attached to a host, gene expression and production of salivary molecules is upregulated, primarily to facilitate feeding and avoid tick rejection by the host. Pathogens exploit tick salivary molecules for their survival and multiplication in the vector and transmission to and establishment in the hosts. Promotion of pathogen transmission by bioactive molecules in tick saliva was described as saliva-assisted transmission (SAT). SAT candidates comprise compounds with anti-haemostatic, anti-inflammatory and immunomodulatory functions, but the molecular mechanisms by which they mediate pathogen transmission are largely unknown. To date only a few tick salivary molecules associated with specific pathogen transmission have been identified and their functions partially elucidated. Advanced molecular techniques are applied in studying tick-host-pathogen interactions and provide information on expression of vector and pathogen genes during pathogen acquisition, establishment and transmission. Understanding the molecular events on the tick-host-pathogen interface may lead to development of new strategies to control tick-borne diseases.

## Introduction

Ticks are obligate blood feeding ectoparasites of a wide range of vertebrates (amphibians, reptiles, birds, mammals). To acquire a blood meal, ticks insert their highly specialized mouthparts through the host skin and, depending on the species, anchor them in the skin by attachment cement (Sonenshine, 1991). Fast feeding soft ticks (Argasidae) feed repeatedly and rapidly with deep penetration of the host skin, causing considerable damage to the host (Binnington and Kemp, 1980), whereas hard ticks (Ixodidae) feed only once in each developmental stage for a prolonged period and penetrate the host epidermis either superficially (Metastriata, e.g., *Dermacentor* spp., *Rhipicephalus* spp.), or more deeply (Prostriata, e.g., *Ixodes* spp., Metastriata, e.g., *Amblyomma* spp.) (Sonenshine, 1991; Bowman et al., 1997a). Ticks are pool feeders; during the process of penetration of the host skin and probing for blood, capillaries and small blood vessels are injured and an extensive haemorrhagic pool forms at the feeding lesion in the host dermis. Hard ticks may require several days to weeks to complete their blood meal. The volume of ingested blood and the duration of feeding are developmental stage- and species-specific, whereby tick females may ingest more blood than 100-times their initial body weight (e.g., Sauer et al., 1995).

**Figure 1:**
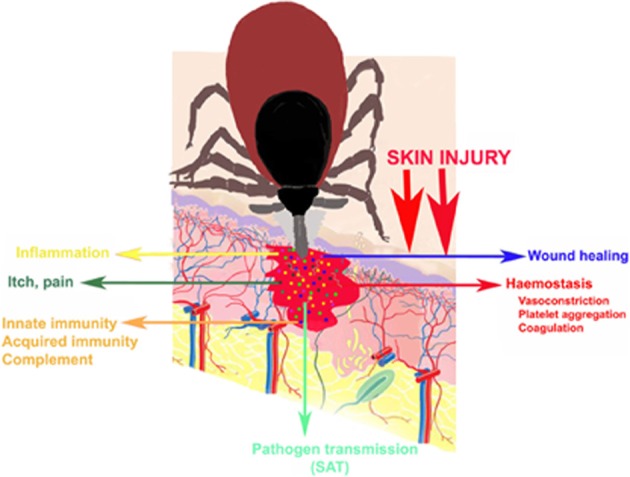
**Hard ticks (Ixodidae) insert their mouthparts into the skin of their hosts and cause tissue injury.** Tick mouthparts are anchored in the host skin by a cement cone. At the tick attachment site, a haemorrhagic pool is created. During the prolonged blood-meal ticks secrete a rich cocktail of bioactive salivary molecules to the host and modulate host defence responses (itch, pain, haemostasis, inflammation, immune reactions) to their benefit. The tick salivary cocktail contains molecules (SAT factors) that facilitate pathogen transmission and infection of the host.

A host would normally react to damage of the skin and the presence of the feeding tick by the formation of a haemostatic plug, activation of the coagulation cascade, vasoconstriction, inflammatory responses leading to wound healing and tissue remodeling, all of which would disrupt tick feeding and cause rejection of the tick, with detrimental effects on tick viability and reproduction. However, ticks succeed in completing their blood meal due to the presence of a large number of biologically active molecules in their salivary glands, displaying anticoagulation, antiplatelet, vasodilatory, anti-inflammatory, and immunomodulatory activities. These molecules have developed during the host-parasite co-evolution and are crucial to overcoming haemostatic and immune responses of the host, enabling ticks to complete feeding and development (Wikel, 1996; Bowman et al., 1997a; Brossard and Wikel, 2008; Nuttall and Labuda, 2008; Francischetti et al., 2009; Mans, 2010; Fontaine et al., 2011). Tick saliva composition is complex and in many cases redundant, reflecting the complex and redundant host defence responses. Some of the tick salivary compounds have been characterized and their functions identified, but the functions remain unknown for most of the molecules (Andrade et al., 2005; Steen et al., 2005; Ribeiro et al., 2006; Brossard and Wikel, 2008; Francischetti et al., 2009; Fontaine et al., 2011) (Figure 1).

In addition to blood feeding, ticks are vectors of a large number of pathogenic microorganisms (viruses, bacteria, protozoa) causing diseases in humans and animals. The common route of a pathogen within the vector is ingestion via infected host blood, migration through the gut to the haemocoel and the penetration of salivary glands. For many pathogens, salivary glands are the organs where they develop and multiply. Thus, tick salivary glands are suggested to play a key role in pathogen transmission to the vertebrate host. However, transmission of pathogens via tick saliva is not a simple mechanistic process, instead pathogens exploit tick salivary molecules for their survival and multiplication in the vector and for transmission to and establishment in the hosts (Bowman et al., 1997a; Ramamoorthi et al., 2005; Brossard and Wikel, 2008; Nuttall and Labuda, 2008).

The phenomenon of promotion of pathogen transmission via arthropod saliva (saliva-assisted transmission, SAT) has been reported in a number of blood-feeding arthropods, including ticks, however, the molecular mechanisms of these processes are largely unknown (Nuttall and Labuda, 2008). Although SAT has been reported for several tick-pathogen associations, only a limited number of tick molecules associated with pathogen transmission have been identified (Ramamoorthi et al., 2005; Hovius et al., 2008b). Therefore, understanding the physiology of tick salivary glands is important for the elucidation of their role in both the modulation of host defences and pathogen transmission.

The molecular background of tick-host associations (e.g., Brossard and Wikel, 2008; Francischetti et al., 2009), their significance in transmission of tick-borne pathogens (e.g., Nuttall and Labuda, 2008) and the natural ecology of tick-host-pathogen interactions with consequences for epidemiology of tick-borne infections in humans (Randolph, 2009; Estrada-Peña et al., 2012) have been extensively reviewed. The present survey summarizes the current knowledge on main tick strategies to overcome host defence responses and the assistance of tick saliva in the transmission of tick-borne pathogens. Although intensive research on tick salivary gland transcriptomes and proteomes is in progress, there are indications that the range of biologically active compounds in tick salivary glands is much wider and gaps exist in understanding their complexity and interactions during the process of feeding and pathogen transmission.

## Tick salivary glands

Tick salivary glands are multifunctional complex organs (Sonenshine, 1991; Sauer et al., 1995, 2000; Bowman and Sauer, 2004; Bowman et al., 2008). In fasting ticks, salivary glands assist in the absorption of water vapor from unsaturated air. They consist of an anterior region of acini (generally agranular and primarily involved in osmo-regulation) attached directly to the main duct. The acini are arranged more caudally in lobules connected by intralobular and interlobular ducts to the main salivary duct. The caudal acini increase greatly in size during feeding and are involved in the production and secretion of salivary bioactive components. The main salivary ducts pass antecranially into the salivarium which fuses with the pharynx and forms the oral cavity. Salivary glands enable the feeding ticks to concentrate blood nutrients by returning excess water and ions via saliva to the host as the ingested host tissues and tick saliva flow in alternate directions through the common buccal canal. The regulation of salivary gland development, degeneration and fluid secretion are under neuro-hormonal control (Bowman and Sauer, 2004; Bowman et al., 2008).

Almost all ixodid ticks produce cement proteins that enable attachment of the tick to the host and seal the area around the mouthparts at the wound site. After a tick attaches to a host, expression of a series of genes and synthesis of proteins is initiated in their salivary glands, which reflect the stages of the feeding process. As feeding progresses, the amount of secreted saliva increases and salivary glands undergo a remarkable and rapid structural reorganization. At the peak of the feeding process, the glands can increase as much as 25-fold in size and content. Once the tick is engorged and detaches, the glands degenerate through a process of cell apoptosis (Bowman et al., 2008).

The composition of tick saliva is complex and redundant in many cases and reflects complex and redundant host defence responses. Tick saliva contains a large number of various non-proteinaceous substances and secreted proteins which are differentially produced during feeding and comprise inhibitors of blood coagulation and platelet aggregation, vasodilatory and immunomodulatory substances as well as compounds preventing itching and pain (Ribeiro et al., 1985; Wikel and Alarcon-Chaidez, 2001; Andrade et al., 2005; Steen et al., 2005; Brossard and Wikel, 2008; Francischetti et al., 2009). The blood-feeding strategy of ticks and, on the other hand, the pool and mode of action of the pharmacologically active compounds contained in their saliva and salivary glands are mostly species-specific. The activity, mechanisms of action and characteristics of these compounds have been studied more intensively during the last two decades and a number of novel molecules have been identified. Some of the tick salivary molecules have pleiotropic effects as they interfere with different arms of the defence responses of the vertebrate hosts. (e.g., Ribeiro and Francischetti, 2003; Francischetti et al., 2009).

Most of the efforts to identify bioactive molecules from ticks are aimed at preparing the active compounds in recombinant form, with their prospective use as pharmaceuticals. In addition, elucidation of the molecular mechanisms of interaction between the ectoparasites and their hosts and of the mechanisms of exploitation of tick molecules by pathogens to invade ticks and hosts can lead to the discovery of new vaccine targets against ticks and the pathogens that ticks transmit (e.g., Willadsen, 2004; Titus et al., 2006; Maritz-Olivier et al., 2007; Hovius et al., 2008b).

## Tick salivary compounds and host haemostasis

Haemostasis is a complex and efficient mechanism that controls blood loss after vascular injury through a series of physiological events leading to termination of blood loss from damaged blood vessels (vasoconstriction), formation of a platelet plug, fibrin clot formation and fibrinolysis (Hoffman et al., 2009).

Research into the mechanisms by which ticks inhibit host haemostasis has led to the discovery and characterization of a variety of compounds with diverse biological activities and potential use in development of novel pharmaceuticals (Kazimírová, 2007; Francischetti et al., 2009; Koh and Kini, 2009; Chmelar et al., 2012). Differences in the anti-haemostatic repertoires suggest that anti-haemostatic mechanisms in hard and soft ticks evolved independently (Mans et al., 2008; Mans, 2010). Saliva of the same tick species simultaneously contain a number of anti-haemostatic molecules, inhibiting different arms of the haemostatic system, or in contrast, the same compounds can display multiple functions (Bowman et al., 1997a; Mans and Neitz, 2004; Valenzuela, 2004; Steen et al., 2005; Maritz-Olivier et al., 2007; Francischetti et al., 2009) (Table 1). However, it is important to note that the cocktail of anti-haemostatic compounds in tick saliva differs between species and in fact, there is no tick species whose complete anti-haemostatic capacities have been fully explored. In addition to discrovery of new sources of drug candidates, studies on tick anti-haemostatics contribute to our understanding of the mechanisms of interactions between ticks and their hosts in the process of feeding and pathogen transmission.

**Table 1:** **Examples of tick salivary molecules that modulate host defence reactions**.

**Tick species**	**Molecule**	**Target and/or function**	**References**
**VASODILATION**			
*Ixodes scapularis*	Prostacyclin	Vasodilation	Ribeiro et al., 1988
*I. scapularis*	tHRF	Vasodilation	Dai et al., 2010
*Ixodes ricinus*	IRS-2	Cathepsin G, chymase	Chmelar et al., 2011, 2012
*Amblyomma americanum*	Prostaglandins	Vasodilation	Bowman et al., 1995
**PLATELET AGGREGATION INHIBITORS**			
Soft ticks (Argasidae)	Apyrase	ATP, ADP	Mans et al., 1998a,b
*Ornithodoros moubata*	Moubatin	Collagen receptor	Waxman and Connolly, 1993
*O. moubata*	Disaggregin	Integrin antagonist	Karczewski et al., 1994
*Ornithodoros savignyi*	Savignygrin	Integrin antagonist	Mans et al., 2002b
*I. scapularis*	Apyrase	ATP, ADP	Ribeiro et al., 1985
*I. scapularis, I. pacificus*	Ixodegrin	Integrin antagonist	Francischetti et al., 2005b
*I. ricinus*	IRS-2	Thrombin	Chmelar et al., 2011
*Haemaphysalis longicornis*	Longicomin	Collagen receptor	Cheng et al., 1999
*Dermacentor variabilis*	Variabilin	Integrin antagonist	Wang et al., 1996
**ANTICOAGULATION AND FIBRINOLYSIS**			
*O. moubata*	Ornithodorin	Thrombin	Van de Locht et al., 1996
*O. moubata*	TAP	FXa	Waxman et al., 1990
*O. savignyi*	Savignin	Thrombin	Nienaber et al., 1999
*O. savignyi*	TAP-like protein	FXa	Joubert et al., 1998
*I. scapularis*	Ixolaris	Tissue factor (TF) pathway inhibitor	Francischetti et al., 2002
*I. scapularis*	Salp 14	Intrinsic pathway	Narasimhan et al., 2002
*I. scapularis*	TIX-5	Inhibitor FXa-mediated FV activation	Schuijt et al., 2013
*I. ricinus*	Ir-CPI	Intrinsic pathway, fibrinolysis	Decrem et al., 2009
*Amblyomma variegatum*	Variegin	Thrombin	Koh et al., 2007
*Amblyomma cajennense*	Amblyomin-X	FXa	Batista et al., 2010
*H. longicornis*	Madanin-1; Madanin-2	Thrombin	Iwanaga et al., 2003
*H. longicornis*	Haemaphysalin	FXII/XIIa	Kato et al., 2005
*H. longicornis*	Longistatin	Fibrinolysis	Anisuzzaman et al., 2011
*Rhipicephalus appendiculatus*	65 kDa protein	Prothrombinase complex	Limo et al., 1991
*Rhipicephalus (Boophilus) microplus*	BmAP	Thrombin	Horn et al., 2000
	Boophilin	Thrombin, trypsin, plasmin	Macedo-Ribeiro et al., 2008
	Microphilin	Thrombin	Ciprandi et al., 2006
*Boophilus calcaratus*	Calcaratin	Thrombin	Motoyashiki et al., 2003
**COMPLEMENT INHIBITORS**			
*O. moubata*	OmCI	C5, prevention of interaction of C5 with C5 convertase	Nunn et al., 2005
*I. scapularis*	Isac	Alternative complement pathway, interacts with C3 convertase	Valenzuela et al., 2000
*I. scapularis*	Salp 20	C3 convertase	Tyson et al., 2007
*I. ricinus*	IRAC I, II, Isac paralogues	Alternative complement pathway, interacts with C3 convertase	Daix et al., 2007
**IMMUNOSUPPRESSANTS/IMMUNOMODULATORS**			
*I. scapularis*	Salp15	Impairs IL-2 production and T cell proliferation; binds *B. burgdorferi* OspC, protects the spirochaete from antibody-mediated killing	Anguita et al., 2002; Ramamoorthi et al., 2005
*I. scapularis*	IL-2 binding protein	Inhibits proliferation of human T cells and CTLL-2 cells	Gillespie et al., 2001
*I. scapularis*	ISL 929 and ISL 1373	Impair adherence of polymorphonuclear leukocytes	Guo et al., 2009
*I. scapularis*	Sialostatin L, L2	Inhibits cathepsin L activity	Kotsyfakis et al., 2006
*I. ricinus*	Iris	Modulates T lymphocyte and macrophage responsiveness, induces Th2 type responses	Leboulle et al., 2002; Prevot et al., 2006
*I. ricinus*	BIP	Inhibitor of B cell proliferation	Hannier et al., 2004
	Ir-LBP	Impairs neutrophil functions	Beaufays et al., 2008
*Dermacentor andersoni*	P36	T cell inhibitor	Bergman et al., 2000
*Hyalomma asiaticum*	BIF	Inhibits LPS-induced proliferation of B cells	Yu et al., 2006
	Hyalomin A, B	Supresses host inflammatory responses (modulation of cytokine secretion, detoxification of free radicals)	Wu et al., 2010
*R. appendiculatus*	Japanin	Reprogrammes DC responses	Preston et al., 2013
*Dermacentor reticulatus*	SHBP	Histamin and serotonin binding protein	Sangamnatdej et al., 2002
*R. appendiculatus*	RaHBP(M), RaHBP(F)	Histamin binding proteins	Paesen et al., 1999
*R. appendiculatus*	TdPI	Tryptase inhibitor	Paesen et al., 2007
*A. americanum*	MIF	Inhibitor of macrophage migration	Jaworski et al., 2001
*R. sanguineus*	Ado, PGE_2_	Modulate host inflammatory responses	Oliveira et al., 2011
**CHEMOKINE BINDING**			
*Rhipicephalus sanguineus*	Evasin-1	Chemokines CCL3, CCL4, CCL18	Frauenschuh et al., 2007; Déruaz et al., 2008
	Evasin-3	Chemokines CXCL8 and CXCL1	
	Evasin 4	Chemokines CCL5 and CCL11	
**WOUND HEALING, ANGIOGENESIS**			
*I. scapularis*	Metalloprotease	Inhibits angiogenesis	Francischetti et al., 2005b
*I. ricinus*	Metalloproteases	Involvement in tissue remodeling or disruption through digestion of structural components	Decrem et al., 2008
*H. longicornis*	Haemangin	Ihibitits angiogenesis	Islam et al., 2009
	HLTnI; troponin I-like molecule	Ihibitits angiogenesis	Fukumoto et al., 2006

*Abbreviatons: tHRF, tick histamine release factor; IRS, I. ricinus serpin; TAP, tick anticoagulant peptide; TIX-5, tick inhibitor of factor Xa toward factor V; Ir-CPI, coagulation contact phase inhibitor from I. ricinus; BmAP, B. microplus anticoagulant protein; SHBP, serotonin- and histamine-binding protein; TdPI, tick-derived peptidase inhibitor; MIF, macrophage migration inhibitory factor; OmCI, O. moubata complement inhibitor; Isac, I. scapularis salivary anticomplement; Irac, I. ricinus anticomplement; Salp, salivary protein; ISL 929 and ISL 1373, I. scapularis salivary proteins 929 and 1373; Iris, I. ricinus immunosuppressor; BIP, B-cell inhibitory protein; P36, 36-kDa immunosuppressant protein; BIF, B-cell inhibitory factor; Ado, adenosine; PGE_2_, prostaglandin E_2_*.

### Vasodilators

Following probing and injury of blood vessels by tick mouthparts, arachidonic acid is released by activated platelets and is converted into thromboxane A_2_, a platelet-aggregating, platelet-degranulating, and vasoconstricting substance. Activated platelets release serotonin which, together with thromboxane A_2_, is responsible for early vasoconstriction in local inflammation caused by tissue injury. To antagonize vasoconstrictors produced by the host at the site of tissue injury, vasodilators are secreted by ticks to the feeding pool. To date, only non-proteinaceous vasodilatory compounds have been identified in tick saliva. These include lipid derivatives such as prostacyclin and prostaglandins (Ribeiro et al., 1988, 1992; Bowman et al., 1996). However, a tick histamine release factor (tHRF), secreted in *Ixodes scapularis* saliva (Dai et al., 2010) and a novel *Ixodes ricinus* serine proteinase inhibitor (serpin) named IRS-2, which inhibits cathepsin G and chymase (Chmelar et al., 2011), probably also act as modulators of vascular permeability (Chmelar et al., 2012).

### Inhibitors of platelet aggregation

Platelet aggregation represents the initial and most immediate stage of haemostasis. Following vascular injury, platelets adhere to the subendothelial tissue and become activated by agonists such as collagen, thrombin, adenosine diphosphate (ADP), and thromboxane A_2_. Agonists bind to specific receptors on the surface of platelets and initiate a long and highly complex chain of intracellular chemical reactions that lead to platelet aggregation and the formation of a heamostatic plug. The platelet aggregation cascade is targeted by ticks at several stages (Francischetti, 2010). A strategy used by a number of ticks is targeting ADP, an agonist important for completion of platelet aggregation, via salivary apyrase. Apyrase, an adenosine triphosphate (ATP)-diphosphohydrolase enzyme, hydrolyses the phosphodiester bonds of ATP and ADP. Apyrase activity has been demonstrated in the salivary glands and saliva of both soft ticks (Ribeiro et al., 1991; Mans et al., 1998a,b, 2002a) and hard ticks (e.g., *I. scapularis;* Ribeiro et al., 1985). Apyrase from *Rhipicephalus (Boophilus) microplus* belongs to the 5′-nucleotidase family (Liyou et al., 1999). On the other hand, apyrase activity has not been detected in the saliva of, e.g., *Amblyomma americanum* (Ribeiro et al., 1992), but increased prostaglandin levels in the saliva of this tick inhibit platelet aggregation by preventing ADP secretion during platelet activation (Ribeiro et al., 1992; Bowman et al., 1995).

Some of the tick-derived platelet aggregation inhibitors interfere with the interaction of collagen with platelet receptors. Activation of platelets by collagen is prevented, e.g., by Moubatin, a specific inhibitor of collagen stimulated platelet activation from *Ornithodoros moubata*, while tick adhesion inhibitor (TAI) identified in the same tick species inhibits the adhesion of platelets to matrix collagen (Waxman and Connolly, 1993; Karczewski et al., 1995). Moubatin belongs to the family of lipocalins and probably prevents platelet aggregation caused by ADP released from collagen-activated platelets (Valenzuela, 2004). Longicornin, another inhibitor of collagen-mediated platelet aggregation, was isolated from the hard tick *Haemapysalis longicornis* (Cheng et al., 1999). However, Longicornin does not bind directly to collagen fibers and does not affect platelet adhesion to collagen, indicating that the inhibitor, similarly to Moubatin, shares a common receptor with collagen.

Thrombin is a key enzyme in thrombosis and haemostasis. In addition to its main role in the formation of the fibrin clot, it induces platelet aggregation. Three functional sites have been recognized in thrombin—the active site, the anion-binding exosite I that mediates binding of thrombin to fibrinogen, the platelet receptor and thrombomodulin, and the anion-binding exosite II, which is the heparin-binding site. Salivary antithrombins detected in both soft ticks and hard ticks that are involved in the inhibition of the coagulation cascade, also inhibit thrombin-induced platelet aggregation (Hoffmann et al., 1991; Nienaber et al., 1999; Kazimírová et al., 2002). The serpin IRS-2 from *I. ricinus* inhibits both cathepsin G- and thrombin-induced platelet aggregation (Chmelar et al., 2011).

Post-activation inhibitors of platelet aggregation target the platelet fibrinogen receptor. Activated platelets express surface adhesion receptor proteins, known as integrins that enable cell-cell and cell-matrix interactions. As the platelets are activated by platelet agonists (thrombin, collagen, and ADP), the ligands [fibronectin, vitronectin, von Willebrand factor, which have the common Arg—Gly—Asp (RGD) sequence, and fibrinogen] bind to glycoprotein (GP)IIb-IIIa via their RGD motif. Interaction between the fibrinogen and the GPIIb-IIIa complex is the important final step in platelet aggregation. Non-RGD disintegrins block the binding of fibrinogen to the integrin αIIbβ3, which is a fibrinogen receptor on surface of activated platelets. The αIIbβ3 antagonists can displace fibrinogen from its receptor thereby allowing disaggregation. Tick-derived disintegrin-like peptides such as Savignygrin (Mans et al., 2002b) and Variabilin (Wang et al., 1996) contain the integrin recognition motif RGD used for binding to GPIIb-IIIa and can inhibit platelet aggregation by preventing the binding of other ligands to the platelet receptor. In contrast, Disaggregin, a fibrinogen receptor antagonist from the soft tick *O. moubata*, is a GPIIb-IIIa antagonist, which lacks the RGD motif and inhibits platelet aggregation by preventing binding to ligands by distinct mechanisms from disintegrin-like peptides (Karczewski et al., 1994). Ixodegrins from *Ixodes pacificus* and *I. scapularis* display sequence similarity to Variabilin, with two additional cystein**e**s in the RGD position (Francischetti et al., 2005b), but their disintegrin activity has yet to be confirmed (Francischetti, 2010).

Disaggregation of platelet aggregates is considered an important back-up mechanism that ticks can use if first-line defence mechanisms fail to inhibit platelet aggregation (Mans and Neitz, 2004). Aggregated platelets may be disaggregated by the removal of fibrinogen from the fibrinogen receptor by competitive binding of an antagonist to the fibrinogen receptor. Proteolysis of fibrinogen can also lead to platelet disaggregation. Apyrase from the soft tick *O. moubata* displays a disaggregation effect on aggregated platelets (Mans et al., 2000), whereas the GPIIb-IIIa antagonist Savignygrin from *O. savignyi* can displace fibrinogen from its receptor and lead to disaggregation (Mans et al., 2002c).

### Inhibitors of the blood-coagulation cascade

Blood coagulation involves a series of enzymatic reactions where an inactive proenzyme (coagulation factor) is converted to an active form, which then activates the next proenzyme in the cascade. Thrombin is involved in the final common pathway of the coagulation cascade and converts fibrinogen into fibrin clots, but also regulates the activity of blood coagulation factors and stimulates platelet reactions. Ticks have evolved powerful tools to prevent or prolong blood coagulation throughout their extended blood meal. A number of inhibitors of serine proteinases that are involved in the coagulation cascade have been identified and characterized from ticks. The majority of inhibitors identified so far are proteins that display a variety of molecular masses, targets and inhibitory mechanisms (Koh and Kini, 2009; Kazimírová et al., 2010). Based on the mechanism of action, anticoagulants from ticks can be classified as: thrombin inhibitors; inhibitors of activated factor X (FXa); inhibitors of the extrinsic tenase complex (ETC); contact system protein inhibitors (Koh and Kini, 2009), with thrombin and FXa being the most common targets.

#### Inhibitors of thrombin

Thrombin inhibitors derived from tick saliva belong to at least seven structural classes and target the enzyme at different sites and via different mechanisms (Koh and Kini, 2009). They comprise mainly the Kunitz-type proteinase inhibitors, Ornithodorin (Van de Locht et al., 1996), Savignin (Nienaber et al., 1999), Boophilin (Macedo-Ribeiro et al., 2008), and Rhipilin (Gao et al., 2011), antithrombins of the hirudin-like/Madanin/Variegin superfamily—Madanin I and II (Iwanaga et al., 2003) and Variegin (Koh et al., 2007), as well as various other peptides not ranked in any of the previous groups, e.g., Microphilin (Ciprandi et al., 2006), *Boophilus microplus* anticoagulant protein (BmAP) (Horn et al., 2000), or Calcaratin (Motoyashiki et al., 2003).

#### Inhibitors of factor Xa

The tick anticoagulant peptide (TAP) from saliva of the soft tick *O. moubata* has been the most intensively studied tick anticoagulant. TAP has some homology with Kunitz type inhibitors, but is a highly specific, reversible competitive inhibitor of FXa (Waxman et al., 1990). The soft tick *O. savignyi* also contains an FXa inhibitor with 46% identity to TAP (Joubert et al., 1998). The recombinant protein Amblyomin-X derived from an *Amblyomma cajennense* transcript encoding a protein containing an N-terminal Kunitz-type domain and a C-terminus with no homology to any known sequences was also found to inhibit FXa (Batista et al., 2010). Salp14, a protein belonging to the salivary protein (Salp) family was identified in saliva of *I. scapularis* and specifically inhibits the FXa active site (Narasimhan et al., 2002, 2004). An unnamed anticoagulant from *Rhipicephalus appendiculatus* saliva probably targets components of the prothrombinase complex, however, its mechanism of action has not been elucidated (Limo et al., 1991).

#### Inhibitors of the extrinsic tenase complex (ETC)

Ixolaris, a two domain Kunitz-type inhibitor of ETC and penthalaris, containing five Kunitz domains, both with homology to the tissue factor (TF) pathway inhibitor, were detected in *I. scapularis* (Francischetti et al., 2002, 2004). Recombinant ixolaris and penthalaris bind to FXa or FX and inhibit the TF/FVIIa complex. Inhibition of factor V and factor VII has been described for salivary gland extracts (SGE) of *Dermacentor andersoni* (Gordon and Allen, 1991), but the compound(s) have not been further characterized.

By screening a yeast surface display library prepared from salivary gands of nymphal *I. scapularis*, a salivary antigen named P23 was identified. Recombinant P23 (rP23) was found to delay the TF initiated thrombin generation (Schuijt et al., 2011b). Further analysis of rP23 (renamed TIX-5, tick inhibitor of factor Xa toward factor V) showed that the protein prolonged activation of the coagulation system by specifically inhibiting the factor Xa-mediated activation of factor V (Schuijt et al., 2013). This study revealed a unique molecular mechanism by which ticks inhibit the coagulation system of heir hosts and, in addition, the results brought new understanding on early activation of blood coagulation. Moreover, immunization with TIX-5 impaired tick feeding, indicating that inhibition of TIX-5 prevents the host anticoagulation mechanism needed for optimal tick feeding.

#### Contact system protein inhibitors

The tick-derived inhibitors of the contact phase described so far belong to the Kunitz-type proteinase inhibitor family. BmTI-A (*B. microplus* trypsin inhibitor-A), inhibits kallikrein and elastase and is present in *B. microplus* larvae (Tanaka et al., 1999). A plasma kallikrein-kinin system inhibitor named Haemaphysalin was identified in *H. longicornis* (Kato et al., 2005). A contact phase inhibitor (Ir-CPI) present in *I. ricinus* salivary glands inhibits the intrinsic coagulation pathway and, to a much lesser extent, fibrinolysis *in vitro* (Decrem et al., 2009).

#### Additional anti-haemostatic activities

Except for antiplatelet factors and anticoagulants, other biological activities which may be related to host haemostasis have been described in the saliva of ticks. Fibrinolytic activity due to the presence of a metalloprotease was detected in saliva of *I. scapularis*. The role of salivary metalloproteinases in tick feeding appears to be related to their antifibrinogen- and antifibrin-specific activities (Francischetti et al., 2003). Kunitz-type serine proteinase inhibitors (RsTI—*Rhipicephalus sanguineus* trypsin inhibitors) were isolated from larvae of *Rhipicephalus sanguineus* (Sant Anna Azzolini et al., 2003). They target plasmin and neutrophil elastase and their role in haemostasis is predicted to be similar to that of serine proteinase inhibitors such as those found, e.g., in *R. (B.) microplus* (see Tanaka et al., 1999). Longistatin, a plasminogen activator identified recently in *H. longicornis* was found to hydrolyse fibrinogen and delay fibrin clot formation (Anisuzzaman et al., 2011).

Several serine protease inhibitors with similarity to proteins of the serpin family were discovered in ticks (Mulenga et al., 2001, 2003). Tick serpins might also interact with host defence responses, including haemostasis. Iris, an immunomodulatory serpin identified in the salivary glands of *I. ricinus* was the first ectoparasite serpin that was reported to both interfere with host haemostasis and the immune response and increase platelet adhesion, the contact phase-activated pathway of coagulation and fibrinolysis (Prevot et al., 2006).

Calcium-binding proteins with sequence homology to the calreticulin family are also present in tick saliva. Tick calreticulins may play a modulating role in host haemostasis through binding calcium ions which are required as coagulation enzyme cofactors (Jaworski et al., 1995). Phospholipase A2, detected in *A. americanum* (Bowman et al., 1997b), is probably responsible for the haemolytic activity of tick saliva.

## Tick salivary compounds and host immune responses

Host cellular innate immune responses and the complement system are the first lines of defence against invading pathogens. Complement comprises a group of serum proteins that can be activated by different pathways. Activation of the complement system leads to the generation of molecules with various biological activities in inflammation and opsonization and lysis of invading pathogens. Adaptive immune response is triggered when activated antigen-presenting cells migrate to lymphoid tissues where they present antigens to T cells, which play a central role in cellular immune responses at the site of infection or assist in the activation of B cells and the generation of an antigen-specific humoral response (Janeway et al., 1999).

Ticks have evolved varius strategies to modulate both innate and acquired immunity of their hosts in order to protect themselves from host immune responses to tick infestation and avoid impaired feeding and/or rejection (Gillespie et al., 2000; Leboulle et al., 2002; Valenzuela, 2004; Brossard and Wikel, 2008) (Table 1). The complex tick-host molecular interactions are considered as a competition between host defences against the ectoparasite and tick evasion strategies. Some hosts develop resistance to repeated tick infestation, while others develop no protective immunity, whereby host resistance or susceptibility depend on the tick-host association and can most likely be explained by tick-induced modulation of the host cytokine network (Andrade et al., 2005; Hajnická et al., 2005).

The *in vitro* effects of saliva and SGE derived from different tick species on functions of host immune effector cells, like granulocytes, macrophages, natural killer (NK) cells, T and B cells, have been extensively documented (e.g., Ramachandra and Wikel, 1992; Kubeš et al., 1994; Ferreira and Silva, 1998; Schoeler et al., 2000a; Gwakisa et al., 2001; Mejri et al., 2002; Hannier et al., 2003). SGE as well as repeated tick infestations are known to suppress the production of pro-inflammatory cytokines and the secretion of Th1 cytokines, whereas they up-regulate Th2 cytokines, indicating a Th2 polarization of the host immune response by ticks (e.g., Ferreira and Silva, 1999; Mejri et al., 2001). Tick-mediated suppression of the Th1 lymphocyte reactivity may inhibit the expansion of antigen-specific T cell clones, differentiation of B cells, activation of macrophages and the NK cell activity. The tick-induced Th2 cytokine profile seems to be advantageous for the survival of the tick because of the anti-inflammatory effect of Th2 cytokines. In addition, the anti-inflammatory mechanisms may also enhance the transmission of tick-borne pathogens (Schoeler and Wikel, 2001; Wikel and Alarcon-Chaidez, 2001).

Despite a relatively broad knowledge of tick-induced host immunomodulation (Brossard and Wikel, 2008), only a limited number of immunomodulatory molecules have been identified and characterized in tick salivary glands (see Table 1). However, deeper understanding of the molecular basis of the strategies used by ticks to evade host resistance and immune mechanisms will probably open new possibilities to design vaccines for tick control and control of the transmission of tick-borne pathogens (Wikel and Alarcon-Chaidez, 2001; Brossard and Wikel, 2008).

### Innate immune responses and complement

Innate immune responses represent the first line of immune defence of the hosts to local injury and involve complement, acute phase proteins, neutrophils, macrophages, mast cells, basophils, eosinophils, dendritic cells (DCs) and NK cells. Complement components, prostaglandins, leukotrienes, chemokines, and cytokines contribute to the recruitment of inflammatory cells to the site of injury (e.g., Andrade et al., 2005). Normally, the consequences of prolonged feeding of an ectoparasite would be local inflammation and rejection. However, ticks produce compounds that inhibit or modulate the pro-inflammatory functions of most cell types infiltrating the attachment site, e.g., neutrophils (Ribeiro et al., 1990; Guo et al., 2009), NK cells (Kubeš et al., 1994), macrophages (Kopecký and Kuthejlová, 1998; Kramer et al., 2011), T cells (e.g., Ramachandra and Wikel, 1992; Bergman et al., 2000) and DCs (Cavassani et al., 2005; Skallová et al., 2008).

The skin is the main organ of the tick-host interface, playing a crucial role in the response of the host to tick infestation as well as in pathogen transmission by the vector. Local modulation of cutaneous immune responses at the tick bite site occurs soon after tick attachment and is characterized by modulation of responses in resident cells that merge into a neutrophil-driven immune response a few hours post-attachment (Heinze et al., 2012a). Ir-LBP, a lipocalin present in *I. ricinus* was shown to inhibit neutrophil chemotaxis *in vitro* and host inflammatory response *in vivo* by decreasing the numbers and activation of neutrophils located at the tick bite site, impairing neutrophil function in inflammation (Beaufays et al., 2008). It was also demonstrated that due to an antialarmin effect on human primary keratinocytes, saliva of *I. ricinus* inhibits cutaneous innate immunity and migration of immune cells to the tick bite site, threreby creating favorable conditions for tick-borne pathogens that are transmitted to and multiply in the host skin (Marchal et al., 2009, 2011).

Recruitment of specific leukocyte populations during the inflammatory response is triggered by chemokines that are key mediators of the inflammatory response against parasites. Ticks have evolved various strategies to manipulate the host cytokine network. The chemokine CXCL8 [interleukin(IL)-8] is a chemo-attractant for neutrophils. Anti-IL-8 activity impairing neutrophil functions was reported from the saliva of various hard ticks (Hajnická et al., 2001). Moreover, tick saliva contains a variety of inhibitory activities directed against other pro-inflammatory cytokines such as IL-2 and the chemokines CCL2 (MCP-1), CCL3 (MIP-1α), CCL5 (RANTES), and CCL11 (eotaxin) (Hajnická et al., 2005). These activities are tick species-, developmental stage-, sex- and feeding stage-specific (Vančová et al., 2010b), but the anti-cytokine factors have not been identified. In contrast, Evasins, a family of novel chemokine binding proteins have been detected in salivary glands of *R. sanguineus* ticks (Frauenschuh et al., 2007). Evasins show selectivity to different chemokines: Evasin-1 binds to CCL3, CCL4, and CCL18; Evasin-3 binds to CXCL8 and CXCL1; and Evasin-4 binds to CCL5 and CCL11 (Frauenschuh et al., 2007; Déruaz et al., 2008). Evasin-3-like activities were also demonstrated for other metastriate tick species, providing further evidence that ticks control host neutrophil functions during feeding (Vančová et al., 2010a). *Hyalomma asiaticum asiaticum* ticks evade host immune reactions by modulating cytokine secretion and detoxification of free radicals (Wu et al., 2010). Two families of immunoregulatory peptides, Hyalomin-A and -B, identified in salivary glands of this species, suppress host inflammatory responses either by inhibiting secretion of tumor necrosis factor (TNF)-alpha, monocyte chemotactic protein-1 (MCP-1), and interferon (IFN)-gamma or by increasing the secretion of the immunosuppressant cytokine IL-10.

Modulation of wound healing and angiogenesis seems to be another strategy used by ticks to suppress host inflammatory responses and succeed in prolonged blood feeding (Francischetti et al., 2009; Hajnická et al., 2011). Tick salivary compounds have been shown to bind the transforming growth factor (TGF)-β1, the platelet-derived growth factor (PDGF), the fibroblast growth factor (FGF)-2 and the hepatocyte growth factor (HGF) in a species-specific manner (Hajnická et al., 2011). *Dermacentor variabilis* saliva suppresses basal and PDGF-stimulated fibroblast migration and reduces extracellular signal-regulated kinase (ERK) activity stimulated with PDGF, suggesting that ticks ensure prolonged maintenance of the feeding lesion in the host skin also by the suppression of ERK activation and fibroblast migration, i.e., important events in wound healing (Kramer et al., 2008).

In addition to growth-factor binding capacities, distinct tick salivary molecules with similarities to disintegrin metalloproteases and thrombospondin are involved in cell-matrix interactions and/or the inhibition of angiogenesis (Valenzuela et al., 2002; Francischetti et al., 2005a; Fukumoto et al., 2006; Harnnoi et al., 2007). The proteins ISL 929 and ISL 1373 with homology to the cysteine-rich domain of disintegrin metalloproteinases which were derived from the sialome of *I. scapularis*, reduce the expression of β2 integrins and impair the adherence of polymorphonuclear leukocytes (PMNs) (Guo et al., 2009). Inhibition of microvascular endothelial cell proliferation by the saliva of *I. scapularis* (Francischetti et al., 2005a) suggests that a metalloprotease is responsible for this activity. In addition to its anticoagulation properties, the Kunitz-like serine proteinase inhibitor Ixolaris from *I. scapularis* downregulates the vascular endothelial growth factor and reduces vessel density in tumours (Carneiro-Lobo et al., 2009). A troponin I-like molecule (HLTnI) present in varius organs, including salivary glands of *H. longicornis*, also inhibits capillary formation of human vascular endothelial cells (Fukumoto et al., 2006) and Haemangin, a Kunitz-type protein from the salivary glands of the same species, displays similar effects on angiogenesis and wound healing (Islam et al., 2009). These results indicate that tick anti-angiogenic factors, in addition to their inhibitory effects on angiogenesis, may also play an important role in controlling tick attachment and pathogen transmission.

Bradykinin and histamine are important mediators of itching and pain and could stimulate host grooming and removal of the feeding ticks. However, ticks developed efficient countermeasures to these host reactions. Tick salivary kininases have been shown to hydrolyse circulating kinins (e.g., bradykinin). For example, a dipeptidyl carboxypeptidase activity was found to account for the kininase activity of *I. scapularis* saliva (Ribeiro and Mather, 1998). In addition, amine-binding proteins of the lipocalin family that suppress host responses to local inflammation are produced by hard ticks. A male-specific histamine-binding salivary protein [RaHBP(M)] and two female-specific histamine-binding salivary proteins [RaHBP(F)-1,2] were isolated from the saliva of *R. appendiculatus* (Ra) (Paesen et al., 1999) and the gene for a protein that binds both serotonin and histamine (SHBP) was identified in *Dermacentor reticulatus* (Sangamnatdej et al., 2002). Recently, a tick derived protease inhibitor (TdPI) has been described and characterized from *R. appendiculatus* (Paesen et al., 2007). TdPI suppresses the activity of human β-tryptases, i.e., mast cell-specific serine proteases with roles in inflammation and tissue remodeling.

Another category of compounds produced by ticks to evade host immune responses are proteins that mimic host proteins. Tick macrophage migration inhibitory factor (MIF) is a peptide detected in salivary glands of the hard tick *Amblyomma americanum* (Jaworski et al., 2001). This peptide inhibits the migration of macrophages and probably protects the feeding ticks from macrophage attack.

Non-proteinaceous substances, like purine nucleoside adenosine (Ado) and prostaglandin PGE_2_ present in saliva of *R. sanguineus*, are also involved in the modulation of host inflammatory and immune responses. These compounds inhibit the production of pro-inflammatory IL-12p40 and TNF-alpha and stimulate the production of anti-inflammatory IL-10 by murine DCs (Oliveira et al., 2011).

The complement system links the innate and adaptive responses of the host immune system and is activated via three main pathways (alternative, classical, and lectin pathway), whereby the alternative pathway is the major line of defence against invading pathogens and is also involved in resistance to ticks. SGE of ixodid ticks were found to inhibit complement activity of vertebrates, whereby the anti-complement activities correlated to the reported host range of the tested tick species (Lawrie et al., 1999). Subsequently, several molecules with anti-complement activities were identified in tick salivary glands. Isac, Salp 20 and Isac-1 from *I. scapularis* (Valenzuela et al., 2000; Tyson et al., 2007) and the Isac paralogues IRAC I and II from *I. ricinus* (Daix et al., 2007; Couvreur et al., 2008) specifically inhibit formation of the C3 convertase of the alternative pathway by blocking binding of complement factor B to complement C3b. On the other hand, OmCI (*Ornithodoros moubata* complement inhibitor) belonging to proteins of the lipocalin family has been the first natural complement inhibitor isolated from a soft tick that specifically targets the C5 activation step in the complement cascade (Nunn et al., 2005).

### Acquired immune responses

During the first exposure to ticks, immunoglobulin and T cell-mediated immune responses are induced in the hosts. Salivary immunogens are processed by Langerhans cells located in the epidermis and presented to immunocompetent lymphocytes (Schoeler and Wikel, 2001; Andrade et al., 2005). Antigen presenting cells can also transport immunogens to draining lymph nodes and promote antibody- and cell-mediated responses. Delayed type hypersensitivity response characteristic of influx of lymphocytes and macrophages, basophils and eosinophils is often observed at the tick feeding site. Homocytotropic antibodies are produced and memory B and T lymphocytes are generated.

Immune resistance to ticks is important in protection from infestation with these ectoparasites and consequently also contributes to the reduction in pathogen transmission from infected ticks to the hosts (Wikel et al., 1997), although specific antigenic and functional components of tick saliva have not been well characterized. In resistant hosts (e.g., rabbits, guinea pigs), the presence of reactive antibodies and effector T lymphocytes assures a rapid response to infestation and can impair tick feeding, whereas ticks have evolved to overcome host immune responses in natural tick-host associations (Ribeiro, 1995). *I. scapularis* salivary antigens that elicit antibodies in resistant hosts have been determined based on screening of salivary gland cDNA expression library with tick-immune mice sera. Using this procedure, the presence of Salp25D, a protein which neutralizes the effect of reactive oxygen species produced by activated neutrophils, has been detected (Das et al., 2001; Narasimhan et al., 2007b). In contrast to resistant hosts, mice generally do not develop acquired resistance to repeated tick feeding (e.g., Schoeler et al., 1999); however, during secondary tick infestation, their cytokine response displays a mixed Th1/Th2 profile and enhanced activity of regulatory T cells (Heinze et al., 2012b).

A variety of tick species have been found to suppress the *in vitro* proliferation of lymphocytes induced with T and/or B cell mitogens. Tick-induced immunosuppression of the host is also characterized by decreased primary antibody responses to T cell-dependent antigens. Moreover, ticks have evolved ways to alter the production of T lymphocyte cytokines. Generally, it has been reported that tick saliva polarizes the host immune response toward a Th2 type profile characterized by the down-regulation of pro-inflammatory Th1 cytokines (IL-2, IFN-gamma) and enhanced production of Th2 cytokines (IL-4, IL-5, IL-6, IL-10, IL-13) (see Gillespie et al., 2000; Schoeler and Wikel, 2001; Wikel and Alarcon-Chaidez, 2001; Brossard and Wikel, 2008, and references therein). It has been suggested that inhibition of T cell responsiveness to mitogens could result from the direct effect of salivary gland proteins on lymphocytes or from their production of IL-10, while up-regulation of IL-4 and IL-10 probably leads to the development of a Th2 response (Ramachandra and Wikel, 1992; Wikel, 1999; Schoeler and Wikel, 2001; Wikel and Alarcon-Chaidez, 2001).

Several T cell inhibitors have been identified in ticks. A 36 kDa protein (P36) suppressing T cell proliferation is present in the saliva of feeding *D. andersoni* (Bergman et al., 2000). Iris was detected in the salivary glands of *I. ricinus* females (Leboulle et al., 2002). The production of Iris is induced in the tick salivary glands during the feeding process and the protein is secreted into the tick saliva. It suppresses T cell proliferation, induces a Th2 type immune response and inhibits the production of pro-inflammatory cytokines IL-6 and TNF-alpha. Salp15, a 15 kDa salivary gland protein from *I. scapularis* is another feeding-induced protein that inhibits the activation of T cells. Salp15 specifically binds to the CD4 molecules on the surface of CD4+ T (helper) cells, which results in inhibition of T cell receptor-mediated signaling, leading to reduced IL-2 production and impaired T cell proliferation (Anguita et al., 2002; Garg et al., 2006). In addition, Salp15 impairs DCs functions by inhibiting Toll-like receptor- and *Borrelia burgdorferi*-induced production of pro-inflammatory cytokines by DCs and DC-induced T cell activation (Hovius et al., 2008a). Evidence was also provided that the pathogen *B. burgdorferi* in *I. scapularis* exploits Salp15 during transmission to a vertebrate host, as it specifically interacts with *B. burgdorferi* outer surface protein C (OspC) and the binding of Salp15 protects *B. burgdorferi* from antibody-mediated killing *in vitro* (Ramamoorthi et al., 2005). Salp 15-like sequences encoding proteins of the Salp family have also been identified in salivary glands of *Ixodes pacificus, I. ricinus*, and *I. persulcatus*, which are other major vectors of disease agents in the USA and Eurasia (Hovius et al., 2007; Hojgaard et al., 2009; Mori et al., 2010). The results suggest that the Salp15 homologues can be involved in host immunomodulation and transmission of *Borrelia* species in the above regions.

Other immunomodulatory proteins facilitating tick feeding and pathogen transmission were also detected in the saliva of *I. scapularis*: a secreted IL-2 binding protein that suppresses T cell proliferation and the activity of immune effector cells responsive to IL-2 stimulation (Gillespie et al., 2001), and the salivary cysteine protease inhibitors sialostatin L and sialostatin L2, with inhibitory activity against cathepsin L (Kotsyfakis et al., 2006). Sialostatin L displays anti-inflammatory properties and inhibits proliferation of cytotoxic T lymphocytes and LPS-induced maturation of DCs, whereas sialostatin L2 does not modulate functions of antigen presenting cells, but is probably important for successful tick feeding (Kotsyfakis et al., 2006; Sá-Nunes et al., 2009). In addition, sialostatin L2 stimulates the growth of *B. burgdorferi* in murine skin, however, the mechanism of this growth stimulation has not been revealed (Kotsyfakis et al., 2010).

Ticks can also benefit from the suppression of B cell responses of vertebrate hosts by inhibiting the production of specific anti-tick antibody responses that could cause rejection of feeding ticks by the host. B cell inhibitory proteins (BIP and BIF) have been identified in *I. ricinus* and *H. asiaticum asiaticum*, respectively (Hannier et al., 2004; Yu et al., 2006). Along with feeding ticks, tick-borne pathogens like *B. burgdorferi* might also benefit from BIP-mediated B cell suppression in their vertebrate hosts (Hannier et al., 2004).

In addition to substances modulating the host immune responses, ticks produce immunoglobulin (IgG)-binding proteins that bind ingested host IgGs and excrete them by salivation. This mechanism protects the ticks primarily from ingested host immunoglobulin's and facilitates their feeding (Wang and Nuttall, 1999).

A novel mechanism of tick-induced modulation of host adaptive immunity which may facilitate pathogen transmission has been discovered recently (Preston et al., 2013). Japanin, a salivary gland protein from *R. appendiculatus* belonging to a new clade of lipocalins from metastriate ticks, was found to target DCs. Japanin specifically reprogramms responses of DCs to a wide variety of stimuli *in vitro*, altering their expression of co-stimulatory and co-inhibitory transmembrane molecules and secretion of pro-inflammatory, anti-inflammatory and T cell polarizing cytokines and it also inhibits the differentiation of DCs from monocytes.

## Tick saliva and its involvement in pathogen transmission

Tick-borne microorganisms are known to exploit tick salivary molecules to increase their pathogenicity and transmission to the vertebrate host, mainly by circumventing host defence responses (Nuttall and Labuda, 2008; Hovius et al., 2008b). In addition, by modulating skin immune reactions, tick saliva enhances non-systemic pathogen transmission between infected and uninfected co-feeding ticks (Labuda et al., 1996).

Exploitation of the highly modified skin site by molecules secreted in tick saliva by tick-borne pathogens has been referred to as SAT, previously saliva-activated transmission, i.e., promotion of transmission of pathogens by vector saliva (Nuttall and Labuda, 2004, 2008).

### Saliva-assisted transmission

The phenomenon of SAT was first described for the Thogoto virus (THOV)—*R. appendiculatus* association. Increased THOV transmission to uninfected *R. appendiculatus* nymphs was observed when the nymphs fed on animals inoculated with a mixture of the virus and SGE of tick females compared to nymphs feeding on animals inoculated with virus only (Jones et al., 1989). Enhanced infection of ticks feeding on animals experimentally inoculated with pathogens and tick saliva (or SGE), i.e., direct evidence of SAT (Nuttall and Labuda, 2008), has subsequently been reported for a few other pathogens, e.g., tick-borne encephalitis virus (TBEV) (Labuda et al., 1993a), *B. burgdorferi* s.l. (Pechová et al., 2002; Zeidner et al., 2002; Macháčková et al., 2006; Horká et al., 2009) and *Francisella tularensis* (Kročová et al., 2003) (see Table 2).

**Table 2:** **Examples of saliva-assisted transmission of tick-borne pathogens**.

**Pathogen**	**Tick species**	**SAT factor, effect**	**References**
THOV	*R. appendiculatus*	SGE, enhanced transmission and infectivity	Jones et al., 1989
TBEV	*I. ricinus*	SGE, enhanced transmission and infectivity	Labuda et al., 1993a
*Borrelia afzelii*	*I. ricinus*	SGE, accelerating effect on spirochaete proliferation in the host, suppression of proinflammatory cytokines	Pechová et al., 2002
*Borrelia burgdorferi* s.s.	*I. ricinus*	SGE, accelerating effect on spirochaete proliferation in the host	Macháčková et al., 2006
*B. burgdorferi* s.s.	*I. ricinus*	Saliva, increased spirochaete load in host skin, increased transmission to ticks	Horká et al., 2009
*Borrelia lusitaniae*	*I. ricinus*	SG lysate, increase of spirochaete loads in target organs	Zeidner et al., 2002
*B. burgdorferi* s.s.	*I. scapularis*	SG lysate, increase of spirochaete loads in target organs	Zeidner et al., 2002
*Francisella tularensis*	*I. ricinus*	SGE, accelerates proliferation of the bacteria in the host	Kročová et al., 2003
THOV	*R. appendiculatus*	Non-viraemic transmission	Jones et al., 1987
TBEV	*I. ricinus*	Non-viraemic transmission	Labuda et al., 1993b
*B. afzelii*	*I. ricinus*	Co-feeding transmission	Richter et al., 2002
*B. burgdorferi* s.s.	*I. ricinus*	Co-feeding transmission	Gern and Rais, 1996
*B. burgdorferi* s.s.	*I. scapularis*	Co-feeding transmission	Piesman and Happ, 2001
TBEV	*I. ricinus*	Saliva, *in vitro* modulation of infection rate of DCs and production of cytokines	Fialová et al., 2010
*B. afzelii*	*I. ricinus*	SGE, anti-inflammatory activities	Severinová et al., 2005
*B. afzelii*	*I. ricinus*	SGE, impairment of signal pathways in DCs	Lieskovská and Kopecký, 2012a,b
		SGE, impairment of DCs functions	Slámová et al., 2011
*B. burgdorferi*	*I. ricinus*	Tick feeding, modulation of skin innate immunity	Kern et al., 2011
	*I. ricinus*	BIP, inhibition of B lymphocyte proliferation induced by the *B. burgdorferi* lipoproteins OspA and OspC	Hannier et al., 2003
*B. burgdorferi*	*I. ricinus*	Salp15 Iric-1, a Salp15 homologue, binds to OspC of *B. burgdorferi* s.s., *B. garinii*, and *B. afzelii*	Hovius et al., 2008c
*B. burgdorferi*	*I. scapularis*	Salp15, immunosuppressive functions, binds to OspC of *B. burgdorferi*, protects the spirochaete from antibody-mediated killing, facilitates transmission and replication of the spirochaete	Ramamoorthi et al., 2005
		Salp25D, antioxidant, facilitates the acquisition of spirochaetes by the vector from an infected mammalian host	Narasimhan et al., 2007b
		Salp20, inhibits complement, facilitates pathogen survival	Tyson et al., 2007
		P8, lectin complement pathway inhibitor, facilitates pathogen transmission	Schuijt et al., 2011a
*A. phagocytophilum*	*I. scapularis*	Salp16, facilitates migration of the pathogen to salivary glands	Sukumaran et al., 2006

In studies involving *Borrelia* spirochaetes, injection of borreliae together with *I. ricinus* or *I. scapularis* SGE increased the level of bacteraemia in the murine host, enhanced the transmission of spirochaetes to feeding ticks and suppressed the production of pro-inflammatory cytokines in draining lymph nodes of mice (Pechová et al., 2002; Zeidner et al., 2002). Moreover, SGE of *I. ricinus* inhibited killing of *B. garinii* by murine macrophages and reduced the production of two major defence molecules of phagocytosis—superoxide and nitric oxide (Kuthejlová et al., 2001). Saliva of *I. scapularis* reduced PMN adhesion via downregulation of beta2-integrins and decreased the efficiency of PMN in the uptake and killing of spirochaetes, thus facilitating the transmission and initial survival of spirochaetes (Montgomery et al., 2004). The SAT compounds responsible for the described effects have not been identified, but they probably depend on vector competence of individual tick species for the pathogen and can vary with different pathogens (Nuttall and Labuda, 2008).

### Non-viraemic transmission

Studies on non-viraemic transmission (NVT) of pathogens from infected to non-infected ticks co-feeding on the same host provide indirect evidence of SAT (Nuttall and Labuda, 2008). By mimicking the natural conditions when infected and non-infected ticks feed in aggregates on a vertebrate host, Jones et al., 1987 demonstrated that transmission of THOV from infected to non-uninfected *R. appendiculatus* ticks co-feeding on non-viraemic guinea pigs was more efficient than transmission on higly viraemic hamsters, and suggested a novel mode of arthropod-borne virus transmission. NVT, independent of a systemic infection of a host, has subsequently been shown for other tick-pathogen associations, mainly TBEV and other arthropod-borne viruses (see Nuttall and Labuda, 2008). Because NVT of TBEV occurs on both susceptible and non-susceptible hosts and can also occur in the presence of virus-specific neutralizing antibodies, it is considered one of the main mechanisms of the maintenance of TBEV in natural foci (Labuda et al., 1993a; Randolph et al., 1999; Randolph, 2011).

Immunomodulation of the tick attachment site by tick salivary compounds is suggested to play a crucial role in the process of NVT as the local skin site of tick attachment is an important focus of viral replication early after transmission (Labuda et al., 1996). It was clearly shown that transmission of TBEV from infected to non-infected *I. ricinus* ticks feeding together on mice was correlated with infection in the skin. The virus was recruited in migratory Langerhans cells and neutrophils preferentially to the site in which ticks were feeding compared with uninfested skin sites and migratory monocyte/macrophages produced infectious virus.

Although the maintenance of the *B. burgdorferi* s.l. spirochaetes depends largely on systemic transmission, transmission of the bacteria between co-feeding ticks has also been demonstrated (Gern and Rais, 1996; Ogden et al., 1997). In laboratory models, duration of infectivity, density and distance between co-feeding ticks have been established as important factors affecting efficiency of transmission of the spirochaetes (Piesman and Happ, 2001; Richter et al., 2002). However, such models seldom mimic the situation in nature. On the other hand, field studies revealed that although sheep do not support systemic infections of *B*. *burgdorferi*, in the absence of alternative hosts, they can transmit localized infections from infected to uninfected ticks co-feeding at the same skin site (Ogden et al., 1997). Due to the presence of various *Borrelia* genospecies and their associations to certain groups of reservoir hosts, the extent and importance of non-systemic transmission in the ecology of Lyme borreliosis needs to be further explored (Randolph et al., 1996; Ogden et al., 1997; Randolph, 2011).

### Host immunomodulation

Immunomodulatory capacities of tick saliva are considered key factors in pathogen transmission. It has been demonstrated that the local skin site infested with ticks and modulated by tick saliva is an important focus of virus replication early after TBEV transmission by ticks. Cellular infiltration and cell migration at the tick attachment site may also facilitate pathogen transmission between infected and uninfected co-feeding ticks (Labuda et al., 1996). These findings were supported by a study where the effects of TBEV infection on DCs and their modulation by *I. ricinus* saliva were demonstrated *in vitro*, showing that treatment of the cells with tick saliva increased the proportion of virus-infected cells and decreased the virus-induced production of TNF-alpha and IL-6 and reduced virus-induced apoptosis (Fialová et al., 2010).

There is increasing evidence that the host immune reactions to *B. burgdorferi* and consequently the outcome of *Borrelia* infection in the host and its infectivity for ticks depend on the presence of the vector. It was demonstrated that mice infected with *B. burgdorferi* via *I. ricinus* were more infective for subsequently attached ticks than those experimentally inoculated with the spirochaete (Gern et al., 1993). BALB/c mice developed a Th2 immune response against *B. burgdorferi* after tick inoculation and a mixed Th1/Th2 response after syringe inoculation. Moreover, in comparison with syringe inoculation of *B. burgdorferi*, IL-4 produced in host draining lymph nodes following tick bites greatly inhibited the production of anti-borrelial IgG2a antibodies (Christe et al., 2000). These findings were further supported by experiments in which a B cell inhibitory protein (BIP) from *I. ricinus* salivary glands suppressed B lymphocyte proliferation induced by the *B. burgdorferi* OspC, suggesting that BIP may play an important role in enhancing *B. burgdorferi* transmission by the tick (Hannier et al., 2003).

Immunomodulation by tick saliva resulting in down-regulation of cytokines, chemokines and antimicrobial peptides in vertebrate hosts was shown to facilitate transmission and infection by *Borrelia*. The significance of the anti-inflammatory properties of *I. ricinus* SGE was demonstrated experimentally in transmission of *B. garinii* to mice when the bacteria were used to stimulate inflammation (Severinová et al., 2005). In these experiments, tick saliva injected together with spirochaetes reduced the numbers of leukocytes and T lymphocytes in the infected murine epidermis at early time-points post infection and decreased the total cell count in draining lymph nodes. Maturation of DCs (Skallová et al., 2008) as well as interactions of *B. garinii* and murine DCs were also impaired by *I. ricinus* saliva through the inhibition of proliferation and IL-2 production by specific CD4+ T cells and decreased production of Th1 and Th2 cytokines by DCs (Slámová et al., 2011). In addition, *I. ricinus* saliva modulated IFN-gamma signaling pathways in DCs (Lieskovská and Kopecký, 2012a) and pathways activated by Toll-like receptor (TLR-2) ligand in *Borrelia*-stimulated DCs (Lieskovská and Kopecký, 2012b).

The involvement of tick salivary compounds in the modulation of skin innate immunity mediated by antimicrobial peptides of the cathelicidin and defensin families in the course of *Borrelia* infection was also demonstrated. When spirochaetes were inoculated to mice, *Borrelia* triggered skin inflammation with induction of the cathelin-related antimicrobial peptide, the mouse cathelicidin and TNF-alpha. However, after natural transmission of the spirochaetes via feeding *I. ricinus*, the inflammatory genes were supressed, suggesting that saliva of the vector tick facilitate *Borrelia* establishment in the host skin (Kern et al., 2011).

Generally, repeated infestation of mice with pathogen-free *Ixodes* ticks results in a polarization of the host immune response toward the Th2 anti-inflammatory cytokine pattern, with a corresponding down-regulation of Th1 responses (Schoeler et al., 1999, 2000b; Mejri et al., 2001). Consequently, down-regulation of pro-inflammatory factors promotes the initial establishment and dissemination of spirochetal infection, but reconstitution of cytokines down-regulated by tick infestation provides protection against tick-transmitted *B. burgdorferi* (Zeidner et al., 1996, 1997). This was demonstrated *in vivo* when mice or Guinea pigs repeatedly infested with pathogen-free *I. scapularis* nymphs were protected against infection with *B. burgdorferi* transmitted via infected ticks, suggesting that immunity against the tick interferes with transmission of the spirochatete (Wikel et al., 1997; Nazario et al., 1998). Moreover, immunization of Guinea pigs with *I. scapularis* salivary gland proteins produced within the first day of tick attachment impaired *B. burgdorferi* transmission from ticks to hosts, probably by evoking acquired immunity against tick feeding (Narasimhan et al., 2007a).

### Exploitation of tick molecules by pathogens

*Borrelia burgdorferi* s.l. displays distinct phenotypic plasticity (Radolf et al., 2012) and exploits a number of tick proteins (Table 2) that support colonization and persistence of the pathogen in the vector and its transmission to the vertebrate host (Kung et al., 2013). Within an infected tick, spirochaetes express OspA and bind to the midgut wall of the tick by using a tick expressed protein (TROSPA) (Pal et al., 2004). After attachment of the tick to a host and onset of feeding, the spirochaetes start to express OspC and move from the midgut through the haemolymph to the salivary glands. In the tick salivary glands, spirochaetes bind to the secreted salivary protein, Salp15, which protects the spirochaetes from antibody-mediated killing and facilitates their transmission and replication in the host skin (Ramamoorthi et al., 2005). The spirochaetes are transmitted to the host with tick saliva containing various salivary molecules which modulate T cells (Salp15), complement (ISAC, Salp20), macrophages, neutrophils, and B cell activities (BIP) and other components of the host immune system (see above) and help *Borrelia* to infect and disseminate in the mammalian host.

Salp15 is the first tick SAT molecule and was identified in salivary glands of *I. scapularis*. Except for immunosuppressive functions, Salp15 is an immunoprotective antigen, because antiserum against the protein protects mice from *Borrelia* infection (Dai et al., 2009).

Salp15 Iric-1, a Salp15 homologue, was identified in *I. ricinus*, the vector of European *Borrelia* species. The protein was found to differentially protect *B. burgdorferi* s.s., *B. garinii*, and *B. afzelii* from antibody-mediated killing in the host (Hovius et al., 2008c).

Salp25D is another immunodominant salivary protein present in *I. scapularis*, which is important during tick acquisition of *B. burgdorferi* and acts as an antioxidant that facilitates pathogen survival (Das et al., 2001; Narasimhan et al., 2007b).

Salp20 protects *B. burgdorferi* from *in vitro* lysis and probably from components of the complement pathway during transmission from an infected tick to the host (Tyson et al., 2007).

A tHRF is up-regulated in *I. scapularis* salivary glands during the rapid feeding phase and probably facilitates tick engorgement and *B. burgdorferi* infection by modulation of vascular permeability and increasing blood flow to the tick bite-site (Dai et al., 2010). Immunization of mice with the recombinant protein interfered with tick feeding and decreased the spirochaete burden.

The Tick Salivary Lectin Pathway Inhibitor (TSLP, formerly P8) from salivary glands of *I. scapularis* was found to interfere with the lectin complement pathway and impair neutrophil phagocytosis and chemotaxis, and protects *Borrelia* from killing by those (Schuijt et al., 2011a).

Tick proteins were identified to be involved also in colonization of the vector and transmission of the intracellular bacterium *Anaplasma phagocytophilum* to vertebrate hosts. Generally, strategies of survival of these bacteria in the vector and transmission to vertebrate hosts are less explored than for *Borrelia. A. phagocytophilum* was found to induce expression of the *I. scapularis* salp16 gene in tick salivary glands during feeding. It was shown that RNA interference-mediated silencing of the salp16 gene expression diminished migration of the bacteria ingested via host blood meal to tick salivary glands, which demonstrates the specific requirement of the pathogen for a tick salivary protein to persist within the vector (Sukumaran et al., 2006). During the transmission of *A*. *phagocytophilum* to the vertebrate host, *I. scapularis* saliva probably modulates host inflammatory responses by inhibition of the production of inflammatory cytokines by macrophages during stimulation of Toll-like (TLR) and Nod-like receptor (NLR) signaling pathways (Chen et al., 2012).

## Conclusions

Ticks have adapted to blood-feeding by counteracting host defence reactions such as haemostasis and immune responses. Ticks modulate host responses at the site of their attachment to the hosts by a wide range of salivary molecules (anti-haemostatics, anti-inflammatory compounds and immunomodulators) and, as a result, they create an environment which is favorable for both the feeding ticks as well as transmission of the microorganisms that ticks may carry. In spite of increasing knowledge on tick salivary compounds primarily involved in modulation of host defences and secondarily in acquisition, survival and transmission of tick-borne microorganisms, large gaps still exist in the identification of the bioactive molecules and characterization of their single or multiple biological functions. In general, there is a large redundancy in tick salivary molecules and, on the other hand, many such molecules can display multiple functions, responding to redundancy in vertebrate defence reactions.

Tick-borne pathogens co-evolved with their vectors and hosts and survive, multiply and circulate due to their adaptation to these different biological systems. Tick salivary molecules, due to their properties, serve as excellent immunomodulators of host immune reactions and as such create a favorable environment to the pathogens that are injected to the host‘s skin together with tick saliva during tick feeding. A wide range of events connected with pathogen transmission to vertebrate hosts facilitated by factors in tick saliva have been described and possible mechanisms of host immunomodulation by tick salivary molecules have been designed. However, the number of identified and characterized tick molecules exploited by pathogens is still limited. Advanced molecular techniques such as DNA microarrays, gene silencing RNA interference, next generation sequencing, *in vitro* studies using tick and host cell cultures, etc. are widely applied in studying tick-host-pathogen interactions. They provide information on the expression of vector and pathogen genes during pathogen acquisition and explain mechanisms of host reactions to the feeding tick and invading microorganisms. Consequently, a deeper understanding of events occurring on the tick-host-pathogen interface may lead to the development of new strategies in the control of tick-borne diseases.

### Conflict of interest statement

The authors declare that the research was conducted in the absence of any commercial or financial relationships that could be construed as a potential conflict of interest.

## References

[B1] Andrade B. B. Teixeira C. R. Barral A. Barral-Netto M. (2005). Haematophagous arthropod saliva and host defense system: a tale of tear and blood. An. Acad. Bras. Ciênc. 77, 665–693 10.1590/S0001-37652005000400008 16341443

[B2] Anguita J. Ramamoorthi N. Hovius J. W. Das S. Thomas V. Persinski R. (2002). Salp15, an *Ixodes scapularis* salivary protein, inhibits CD4(+) T cell activation. Immunity 16, 849–859 10.1016/S1074-7613(02)00325-4 12121666

[B3] Anisuzzaman, Islam M. K. Alim M. A. Miyoshi T. Hatta T. Yamajim K. (2011). Longistatin, a plasminogen activator, is key to the availability of blood-meals for ixodid ticks. PLoS Pathog. 7:e1001312 10.1371/journal.ppat.1001312 21423674 PMC3053353

[B4] Batista I. F. C. Ramos O. H. P. Ventura J. S. Junqueira-de-Azevedo I. L. M. Ho P. L. Chudzinski-Tavassi A. M. (2010). A new Factor Xa inhibitor from *Amblyomma cajennense* with a unique domain composition. Arch. Biochem. Biophys. 493, 151–156 10.1016/j.abb.2009.10.009 19853573

[B5] Beaufays J. Adam B. Menten-Dedoyart C. Fievez L. Grosjean A. Decrem Y. (2008). Ir-LBP, an *Ixodes ricinus* tick salivary LTB4-binding lipocalin, interferes with host neutrophil function. PLoS ONE 3:e3987 10.1371/journal.pone.0003987 19096526 PMC2600610

[B6] Bergman D. K. Palmer M. J. Caimano M. J. Radolf J. D. Wikel S. K. (2000). Isolation and molecular cloning of a secreted immunomosuppressant protein from *Dermacentor andersoni* salivary gland. J. Parasitol. 86, 516–525 10864249 10.1645/0022-3395(2000)086[0516:IAMCOA]2.0.CO;2

[B7] Binnington K. C. Kemp D. H. (1980). Role of tick salivary glands in feeding and disease transmission. Adv. Parasitol. 18, 315–339 10.1016/S0065-308X(08)60403-0 6776790

[B8] Bowman A. S. Ball A. Sauer J. R. (2008). Tick salivary glands: the physiology of tick water balance and their role in pathogen trafficking and transmission, in Ticks: Biology, Disease and Control, eds Bowman A. S. Nuttall P. A. (Cambridge; New York, NY: Cambridge University Press), 73–91 10.1017/CBO9780511551802.004

[B9] Bowman A. S. Coons L. B. Needham G. R. Sauer J. R. (1997a). Tick saliva: recent advances and implications for vector competence. Med. Vet. Entomol. 11, 277–285 10.1111/j.1365-2915.1997.tb00407.x 9330260

[B10] Bowman A. S. Gengler C. L. Surdick M. R. Zhu K. Essenberg R. C. Sauer J. R. (1997b). A novel phospholipase A_2_ activity in saliva of the lone star tick, *Amblyomma americanum* (L.). Exp. Parasitol. 87, 121–132 10.1006/expr.1997.4201 9326887

[B11] Bowman A. S. Dillwith J. W. Sauer J. R. (1996). Tick salivary prostaglandins: presence, origin and significance. Parasitol. Today 12, 388–396 10.1016/0169-4758(96)10061-2 15275289

[B12] Bowman A. S. Sauer J. R. (2004). Tick salivary glands: function, physiology and future. Parasitology 129, S67–S81 10.1017/S0031182004006468 15938505

[B13] Bowman A. S. Sauer J. R. Zhu K. Dillwith J. W. (1995). Biosynthesis of salivary prostaglandins in the lone star tick, *Amblyomma americanum*. Insect Biochem. Mol. Biol. 25, 735–741 10.1016/0965-1748(95)00013-L 7627205

[B14] Brossard M. Wikel S. K. (2008). Tick immunobiology, in Ticks: Biology, Disease and Control, eds Bowman A. S. Nuttall P. A. (Cambridge; New York, NY: Cambridge University Press), 186–204 10.1017/CBO9780511551802.010

[B15] Carneiro-Lobo T. C. Konig S. Machado D. E. Nasciutti L. E. Forni M. F. Francischetti I. M. (2009). Ixolaris, a tissue factor inhibitor, blocks primary tumor growth and angiogenesis in a glioblastoma model. J. Thromb. Haemost. 7, 1855–1864 10.1111/j.1538-7836.2009.03553.x 19624457 PMC2896491

[B16] Cavassani K. A. Aliberti J. C. Dias A. R. Silva J. S. Ferreira B. R. (2005). Tick saliva inhibits differentiation, maturation and function of murine bonemarrow-derived dendritic cells. Immunology 114, 235–245 10.1111/j.1365-2567.2004.02079.x 15667568 PMC1782083

[B17] Chen G. Severo M. S. Sohail M. Sakhon O. S. Wikel S. K. Kotsyfakis M. (2012). *Ixodes scapularis* saliva mitigates inflammatory cytokine secretion during *Anaplasma phagocytophilum* stimulation of immune cells. Parasit. Vectors 5, 229 10.1186/1756-3305-5-229 23050849 PMC3503595

[B18] Cheng Y. Wu H. Li D. (1999). An inhibitor selective for colagen-stimulated platelet aggregation from the salivary glands of hard tick *Haemaphysalis longicornis* and its mechanism of action. Sci. China C Life Sci. 42, 457–464 10.1007/BF02881768 18726508

[B19] Chmelar J. Calvo E. Pedra J. H. F. Francischetti I. M. B. Kotsyfakis M. (2012). Tick salivary secretion as a source of antihemostatics. J. Proteomics 75, 3842–3854 10.1016/j.jprot.2012.04.026 22564820 PMC3383439

[B20] Chmelar J. Oliveira C. J. Rezacova P. Francischetti I. M. Kovarova Z. Pejler G. (2011). A tick salivary protein targets cathepsin G and chymase and inhibits host inflammation and platelet aggregation. Blood 117, 736–744 10.1182/blood-2010-06-293241 20940421 PMC3031492

[B21] Christe M. Rutti B. Brossard M. (2000). Cytokines (IL-4 and IFN-γ) and antibodies (IgE and IgG2a) produced in mice infected with *Borrelia burgdorferi* sensu stricto via nymphs of *Ixodes ricinus* ticks or syringe inoculations. Parasitol. Res. 86, 491–496 10.1007/s004360050699 10894476

[B22] Ciprandi A. de Oliveira S. K. Masuda A. Horn F. Termignoni C. (2006). *Boophilus microplus*: its saliva contains microphilin, a small thrombin inhibitor. Exp. Parasitol. 114, 40–46 10.1016/j.exppara.2006.02.010 16600217

[B23] Couvreur B. Beaufays J. Charon C. Lahaye K. Gensale F. Denis V. (2008). Variability and action mechanism of a family of anticomplement proteins in *Ixodes ricinus*. PLoS ONE 3:e1400 10.1371/journal.pone.0001400 18167559 PMC2151134

[B24] Dai J. Narasimhan S. Zhang L. Liu L. Wang P. Fikrig E. (2010). Tick histamine release factor is critical for *Ixodes scapularis* engorgement and transmission of the lyme disease agent. PLoS Pathog. 6:e1001205 10.1371/journal.ppat.1001205 21124826 PMC2991271

[B25] Dai J. Wang P. Adusumilli S. Booth C. J. Narasimhan S. Anguita J. (2009). Antibodies against a tick protein, Salp15, protect mice from the *Lyme* disease agent. Cell Host Microbe 6, 482–492 10.1016/j.chom.2009.10.006 19917502 PMC2843562

[B26] Daix V. Schroeder H. Praet N. Georgin J.-P. Chiappino I. Gillet L. (2007). *Ixodes* ticks belonging to the *Ixodes ricinus* complex encode a family of anticomplement proteins. Insect Mol. Biol. 16, 155–166 10.1111/j.1365-2583.2006.00710.x 17298559

[B27] Das S. Banerjee G. DePonte K. Marcantonio N. Kantor F. S. Fikrig E. (2001). Salp25D, an *Ixodes scapularis* antioxidant, is 1 of 14 immunodominant antigens in engorged tick salivary glands. J. Infect. Dis. 184, 1056–1064 10.1086/323351 11574922

[B28] Decrem Y. Beaufays J. Blasioli V. Lahaye K. Brossard M. Vanhamme L. (2008). A family of putative metalloproteases in the salivary glands of the tick *Ixodes ricinus*. FEBS J. 275, 1485–1499 10.1111/j.1742-4658.2008.06308.x 18279375

[B29] Decrem Y. Rath G. Blasioli V. Cauchie P. Robert S. Beaufays J. (2009). Ir-CPI, a coagulation contact phase inhibitor from the tick *Ixodes ricinus*, inhibits thrombus formation without impairing hemostasis. J. Exp. Med. 206, 2381–2395 10.1084/jem.20091007 19808248 PMC2768864

[B30] Déruaz M. Frauenschuh A. Alessandri A. L. Dias J. M. Coelho F. M. Russo R. C. (2008). Ticks produce highly selective chemokine binding proteins with antiinflammatory activity. J. Exp. Med. 205, 2019–2031 10.1084/jem.20072689 18678732 PMC2526197

[B32] Estrada-Peña A. Ayllón N. de la Fuente J. (2012). Impact of climate trends on tick-borne pathogen transmission. Front. Physiol. 3:64 10.3389/fphys.2012.00064 22470348 PMC3313475

[B33] Ferreira B. R. Silva J. S. (1998). Saliva of *Rhipicephalus sanguineus* tick impairs T cell proliferation and IFN-g-induced macrophage microbicidal activity. Vet. Immunol. Immunopathol. 64, 279–293 10.1016/S0165-2427(98)00135-4 9730222

[B34] Ferreira B. R. Silva J. S. (1999). Successive tick infestations selectively promote a T-helper 2 cytokine profile in mice. Immunology 96, 434–439 10.1046/j.1365-2567.1999.00683.x 10233725 PMC2326753

[B35] Fialová A. Cimburek Z. Iezzi G. Kopecký J. (2010). *Ixodes ricinus* tick saliva modulates tick-borne encephalitis virus infection ofdendritic cells. Microbes Infect. 12, 580–585 10.1016/j.micinf.2010.03.015 20381639

[B36] Fontaine A. Diouf I. Bakkali N. Missé D. Pagès F. Fusai T. (2011). Implication of haematophagous arthropod salivary proteins in host-vector interactions. Parasit. Vectors 4, 187 10.1186/1756-3305-4-187 21951834 PMC3197560

[B37] Francischetti I. M. B. (2010). Platelet aggregation inhibitors from hematophagous animals. Toxicon 56, 1130–1144 10.1016/j.toxicon.2009.12.003 20035779 PMC2888830

[B38] Francischetti I. M. B. Mather T. N. Ribeiro J. M. C. (2003). Cloning of a salivary gland metalloprotease and characterisation of gelatinase and fibrin(ogen)lytic activities in the saliva of the lyme disease tick vector *Ixodes scapularis*. Biochem. Biophys. Res. Commun. 305, 869–875 10.1016/S0006-291X(03)00857-X 12767911 PMC2903890

[B39] Francischetti I. M. B. Mather T. N. Ribeiro J. M. C. (2004). Penthalaris, a novel recombinant five-Kunitz tissue factor pathway inhibitor (TFPI) from the salivary gland of the tick vector of *Lyme* disease, *Ixodes scapularis*. Thromb. Haemost. 91, 886–898 15116248 10.1160/TH03-11-0715

[B40] Francischetti I. M. B. Mather T. N. Ribeiro J. M. C. (2005a). Tick saliva is a potent inhibitor of endothelial cell proliferation and angiogenesis. Thromb. Haemost. 94, 167–174 16113800 10.1267/THRO05010167 PMC2893037

[B41] Francischetti I. M. B. Pham V. M. Mans B. J. Andersen J. F. Mather T. N. Lane R. S. (2005b). The transcriptome of the salivary glands of the female western black-legged tick *Ixodes pacificus* (*Acari: Ixodidae*). Insect Biochem. Mol. Biol. 35, 1142–1161 10.1016/j.ibmb.2005.05.007 16102420 PMC2887698

[B42] Francischetti I. M. B. Sá-Nunes A. Mans B. J. Santos I. M. Ribeiro J. M. C. (2009). The role of saliva in tick feeding. Front. Biosci. 14, 2051–2088 19273185 10.2741/3363 PMC2785505

[B43] Francischetti I. M. B. Valenzuela J. G. Andersen J. F. Mather T. N. Ribeiro J. M. C. (2002). Ixolaris, a novel recombinant tissue factor pathway inhibitor (TFPI) from the salivary gland of the tick, *Ixodes scapularis*: identification of factor X and factor Xa as scaffolds for the inhibition of factor VIIa/Tissue factor complex. Hemost. Thromb. Vasc. Biol. 99, 3602–3612 11986214 10.1182/blood-2001-12-0237

[B44] Frauenschuh A. Power C. A. Déruaz M. Ferreira B. R. da Silva J. Teixeira M. M. (2007). Molecular cloning and characterization of a highly selective chemokine-binding protein from the tick *Rhipicephalus sanguineus*. J. Biol. Chem. 282, 27250–27258 10.1074/jbc.M704706200 17640866

[B45] Fukumoto S. Sakaguchi T. You M. Xuan X. Fujisaki K. (2006). Tick troponin I-like molecule is a potent inhibitor for angiogenesis. Microvasc. Res. 71, 218–221 10.1016/j.mvr.2006.02.003 16631826

[B46] Gao X. Shi L. Zhou Y. Cao J. Zhang H. Zhou J. (2011). Characterization of the anticoagulant protein Rhipilin-1 from the *Rhipicephalus haemaphysaloides* tick. J. Insect Physiol. 57, 339–343 10.1016/j.jinsphys.2010.12.001 21147114

[B47] Garg R. Juncadella I. J. Ramamoorthi N. Ashish Ananthanarayanan S. K. Thomas V. Rincón M. (2006). Cutting edge: CD4 is the receptor for the tick saliva immunosuppressor, Salp15. J. Immunol. 177, 6579–6583 17082567 10.4049/jimmunol.177.10.6579 PMC4302324

[B48] Gern L. Rais O. (1996). Efficient transmission of *Borrelia burgdorferi* between cofeeding *Ixodes ricinus* ticks (*Acari: Ixodidae*). J. Med. Entomol. 33, 189–192 8906929 10.1093/jmedent/33.1.189

[B49] Gern L. Schaible U. E. Simon M. M. (1993). Mode of inoculation of the *Lyme* disease agent *Borrelia burgdorferi* influences infection and immune responses in inbred strains of mice. J. Infect. Dis. 167, 971–975 10.1093/infdis/167.4.971 8450262

[B50] Gillespie R. D. Dolan M. C. Piesman J. Titus R. G. (2001). Identification of an IL-2 binding protein in the saliva of the *Lyme* disease vector tick, *Ixodes scapularis*. J. Immunol. 166, 4319–4326 11254684 10.4049/jimmunol.166.7.4319

[B51] Gillespie R. D. Lamine Mbow M. Titus R. G. (2000). The immunomodulatory factors of bloodfeeding arthropod saliva. Parasite Immunol. 22, 319–331 10.1046/j.1365-3024.2000.00309.x 10886716

[B52] Gordon J. R. Allen J. R. (1991). Factors V and VII anticoagulant activities in the salivary glands of feeding *Dermacentor andersoni* ticks. J. Parasitol. 77, 167–170 10.2307/3282577 1992089

[B53] Guo X. Booth C. J. Paley M. A. Wang X. DePonte K. Fikrig E. (2009). Inhibition of neutrophil function by two tick salivary proteins. Infect. Immun. 77, 2320–2329 10.1128/IAI.01507-08 19332533 PMC2687334

[B54] Gwakisa P. Yoshihara K. Thanh Long T. Gotoh H. Amano F. Momotani E. (2001). Salivary gland extract of *Rhipicephalus appendiculatus* ticks inhibits *in vitro* transcription and secretion of cytokines and production of nitric oxide by LPS-stimulated JA-4 cells. Vet. Parasitol. 99, 53–61 10.1016/S0304-4017(01)00445-9 11445155

[B55] Hajnická V. Kocáková P. Sláviková M. Slovák M. Gašperík J. Fuchsberger N. (2001). Anti-interleukin-8 activity of tick salivary gland extracts. Parasite Immunol. 23, 483–489 10.1046/j.1365-3024.2001.00403.x 11589777

[B56] Hajnická V. Vančová I. Kocáková P. Slovák M. Gašperík J. Sláviková M. (2005). Manipulation of host cytokine network by ticks: a potential gateway for pathogen transmission. Parasitology 130, 333–342 10.1017/S0031182004006535 15796016

[B57] Hajnická V. Vančová-Štibrániová I. Slovák M. Kocáková P. Nuttall P. A. (2011). Ixodid tick salivary gland products target host wound healing growth factors. Int. J. Parasitol. 41, 213–223 10.1016/j.ijpara.2010.09.005 20934428

[B58] Hannier S. Liversidge J. Sternberg J. M. Bowman A. S. (2003). *Ixodes ricinus* tick salivary gland extract inhibits IL-10 secretion and CD69 expression by mitogen-stimulated murine splenocytes and induces hyporesponsiveness in B lymphocytes. Parasite Immunol. 25, 27–37 10.1046/j.1365-3024.2003.00605.x 12753435

[B59] Hannier S. Liversidge J. Sternberg J. M. Bowman A. S. (2004). Characterization of the B-cell inhibitory protein factor in *Ixodes ricinus* tick saliva: a potential role in enhanced *Borrelia burgdoferi* transmission. Immunology 113, 401–408 10.1111/j.1365-2567.2004.01975.x 15500628 PMC1782588

[B60] Harnnoi T. Sakaguchi T. Nishikawa Y. Xuan X. Fujisaki K. (2007). Molecular characterization and comparative study of 6 salivary gland metalloproteases from the hard tick, *Haemaphysalis longicornis*. Comp. Biochem. Physiol. B Biochem. Mol. Biol. 147, 93–101 10.1016/j.cbpb.2006.12.008 17292650

[B61] Heinze D. M. Carmical J. R. Aronson J. F. Thangamani S. (2012a). Early immunologic events at the tick-host interface. PLoS ONE 7:e47301 10.1371/journal.pone.0047301 23077588 PMC3471850

[B62] Heinze D. M. Wikel S. K. Thangamani S. Alarcon-Chaidez F. J. (2012b). Transcriptional profiling of the murine cutaneous response during initial and subsequent infestations with *Ixodes scapularis* nymphs. Parasit. Vectors 5, 26 10.1186/1756-3305-5-26 22309607 PMC3293053

[B63] Hoffman R. Benz E. J. Furie B. Shattil S. J. (2009). Hematology: Basic Principles and Practice. New York, Edinburgh; London Philadelphia; San Francisco, CA: Churchill Livingstone/Elsevier

[B64] Hoffmann A. Walsmann P. Riesener G. Paintz M. Markwardt F. (1991). Isolation and characterization of a thrombin inhibitor from the tick *Ixodes ricinus*. Pharmazie 46, 209–212 1881945

[B65a] Hojgaard A. Biketov S. F. Shtannikov A. V. Zeidner N. S. Piesman J. (2009). Molecular identification of Salp15, a key salivary gland protein in the transmission of Lyme disease spirochetes, from *Ixodes persulcatus and Ixodes pacificus* (Acari: Ixodidae). J. Med. Entomol. 46, 1458–1463 10.1603/033.046.0631 19960697

[B65] Horká H. Cerná-Kýcková K. Skallová A. Kopecký J. (2009). Tick saliva affects both proliferation and distribution of *Borrelia burgdorferi* spirochetes in mouse organs and increases transmission of spirochetes to ticks. Int. J. Med. Microbiol. 299, 373–380 10.1016/j.ijmm.2008.10.009 19147403

[B66] Horn F. Coutinho dos Carlos P. Termignoni C. (2000). *Boophilus microplus* anticoagulant protein: an antithrombin inhibitor isolated from the cattle tick saliva. Arch. Biochem. Biophys. 384, 68–73 10.1006/abbi.2000.2076 11147837

[B67] Hovius J. W. R. de Jong M. A. W. P. den Dunnen J. Litjens M. Fikrig E. (2008a). Salp15 binding to DC-SIGN inhibits cytokine expression by impairing both nucleosome remodeling and mRNA stabilization. PLoS Pathog. 4:e31 10.1371/journal.ppat.0040031 18282094 PMC2242833

[B68] Hovius J. W. R. Levi M. Fikrig E. (2008b). Salivating for knowledge: potential pharmacological agents in tick saliva. PLoS Med. 5:e43 10.1371/journal.pmed.0050043 18271624 PMC2235897

[B69] Hovius J. W. Schuijt T. J. de Groot K. A. Roelofs J. J. T. H. Oei A. Marquart J. A. (2008c). Preferential protection of *Borrelia burgdorferi* sensu stricto by a Salp15 homologue in *Ixodes ricinus* saliva. J. Infect. Dis. 198, 1189–1197 10.1086/591917 18752445 PMC4317250

[B70] Hovius J. W. Ramamoorthi N. Van't Veer C. de Groot K. A. Nijhof A. M. Jongejan F. (2007). Identification of Salp15 homologues in *Ixodes ricinus* ticks. Vector Borne Zoonotic Dis. 7, 296–303 10.1089/vbz.2006.0624 17896872

[B71] Islam M. K. Tsuji N. Miyoshi T. Alim M. A. Huang X. Hatta T. (2009). The Kunitz-like modulatory protein haemangin is vital for hard tick blood-feeding success. PLoS Pathog. 5:e1000497 10.1371/journal.ppat.1000497 19593376 PMC2701603

[B72] Iwanaga S. Okada M. Isawa H. Morita A. Yuda M. Chinzei Y. (2003). Identification and characterisation of novel salivary thrombin inhibitors from the ixodidae tick, *Haemaphysalis longicornis*. Eur. J. Biochem. 270, 1926–1934 10.1046/j.1432-1033.2003.03560.x 12709051

[B73] Janeway C. A. Travers P. Walport M. Capra J. D. (1999). ImmunoBiology. The Immune System in Health and Disease. 4th Edn., London, NY: Elsevier Science Ltd/Garland Publishing

[B74] Jaworski D. C. Jasinskas A. Metz C. N. Bucala R. Barbour A. G. (2001). Identification and characterization of a homologue of the proinflammatory cytokine, macrophage migration inhibitory factor in the tick, *Amblyomma americanum*. Insect Mol. Biol. 10, 323–331 10.1046/j.0962-1075.2001.00271.x 11520355

[B75] Jaworski D. C. Simmen F. A. Lamoreaux W. Coons L. B. Muller M. T. Needham G. R. (1995). A secreted calreticulin protein in ixodid tick (*Amblyomma americanum*) saliva. J. Insect Physiol. 41, 369–375 10.1016/0022-1910(94)00107-R

[B76] Jones L. D. Davies C. R. Steele G. M. Nuttall P. A. (1987). A novel mode of arbovirus transmission involving a nonviraemic host. Science 237, 775–777 10.1126/science.3616608 3616608

[B77] Jones L. D. Hodgson E. Nuttall P. A. (1989). Enhancement of virus transmission by tick salivary glands. J. Gen. Virol. 70, 1895–1898 10.1099/0022-1317-70-7-1895 2544668

[B78] Joubert A. M. Louw A. I. Joubert F. Neitz A. W. H. (1998). Cloning, nucleotide sequence and expression of the gene encoding factor Xa inhibitor from the salivary glands of the tick, Ornithodoros savignyi. Exp. Appl. Acarol. 22, 603–619 10.1023/A:1006198713791 9890144

[B79] Karczewski J. Endris R. Connolly T. M. (1994). Disagregin is a fibrinogen receptor antagonist lacking the Arg-Gly-Asp sequence from the tick, *Ornithodoros moubata*. J. Biol. Chem. 269, 6702–6708 8120028

[B80] Karczewski J. Waxman L. Endris R. G. Connolly T. M. (1995). An inhibitor from the argasid tick *Ornithodoros moubata* of cell adhesion to collagen. Biochem. Biophys. Res. Commun. 208, 532–541 10.1006/bbrc.1995.1371 7695604

[B81] Kato N. Iwananga S. Okayama T. Isawa H. Yuda M. Chinzei Y. (2005). Identification and characterization of the plasma kallikrein-kinin system inhibitor, haemaphysalin, from hard tick, *Haemaphysalis longicornis*. Thromb. Haemost. 93, 359–367 15711755 10.1160/TH04-05-0319

[B82] Kaufman R. (2010). Ticks: physiological aspects with implications for patogen transmission. Ticks Tick Borne Dis. 1, 11–22 10.1016/j.ttbdis.2009.12.001 21771507

[B83] Kazimírová M. (2007). Bioactive compounds in ticks acting on host thrombohemostasis, in Thrombohemostatic Disease Research, ed Wiwanitkit V. (New York, NY: Nova Science Publishers, Inc.), 95–113

[B84] Kazimírová M. Jančinová V. Petríková M. Takáè P. Labuda M. Nosál' R. (2002). An inhibitor of thrombin-stimulated blood platelet aggregation from the salivary glands of the hard tick *Amblyomma variegatum* (*Acari: Ixodidae*). Exp. Appl. Acarol. 28, 97–105 10.1023/A:1025398100044 14570120

[B85] Kazimírová M. Koh C. Y. Kini R. M. (2010). Tiny ticks are vast sources of antohaemostatic factors, in Toxins and Hemostasis, eds Kini R. M. Clementson K. J. Markland F. S. McLane A. A. Morita T. (Dordrecht; New York, NY: Springer), 113–130 10.1007/978-90-481-9295-3_8

[B86] Kern A. Collin E. Barthel C. Michel C. Jaulhac B. Boulanger N. (2011). Tick saliva represses innate immunity and cutaneous inflammation in a murine model of lyme disease. Vector Borne Zoonotic Dis. 11, 1343–1350 10.1089/vbz.2010.0197 21612525

[B87] Koh C. Y. Kazimirova M. Trimnell A. Takac P. Labuda M. Nuttall P. A. (2007). Variegin, a novel fast and tight binding thrombin inhibitor from the tropical bont tick. J. Biol. Chem. 282, 29101–29113 10.1074/jbc.M705600200 17684009

[B88] Koh C. Y. Kini R. M. (2009). Molecular diversity of anticoagulants from haematophagous animals. Thromb. Haemost. 102, 437–453 19718463 10.1160/TH09-04-0221

[B89] Kopecký J. Kuthejlová M. (1998). Suppressive effect of *Ixodes ricinus* salivary gland extract on mechanisms of natural immunity *in vitro*. Parasite Immunol. 20, 169–174 9618727

[B90] Kotsyfakis M. Horka H. Salat J. Andersen F. F. (2010). The crystal structures of two salivary cystatins from the tick *Ixodes scapularis* and the effect of these inhibitors on the establishment of *Borrelia burgdorferi* infection in a murine model. Mol. Microbiol. 77, 456–470 10.1111/j.1365-2958.2010.07220.x 20545851 PMC2909360

[B91] Kotsyfakis M. Sa-Nunes A. Francischetti I. M. Mather T. N. Andersen J. F. Ribeiro J. M. C. (2006). Antiinflammatory and immunosuppressive activity of sialostatin L, a salivary cystatin from the tick *Ixodes scapularis*. J. Biol. Chem. 281, 26298–26307 10.1074/jbc.M513010200 16772304

[B92] Kramer C. Nahmias Z. Norman D. D. Mulvihill T. A. Coons L. B. Cole J. A. (2008). *Dermacentor variabilis*: regulation of fi broblast migration by tick salivary gland extract and saliva. Exp. Parasitol. 119, 391–397 10.1016/j.exppara.2008.04.005 18492598

[B93] Kramer C. D. Poole N. M. Coons L. B. Cole J. A. (2011). Tick saliva regulates migration, phagocytosis, and gene expression in the macrophage-like cell line, IC-21. Exp. Parasitol. 127, 665–671 10.1016/j.exppara.2010.11.012 21145320

[B94] Kročová Z. Macela A. Hernychová L. Kroca M. Pechová J. Kopecký J. (2003). Tick salivary gland extract accelerates proliferation of *Franciscella tularensis* in the host. J. Parasitol. 89, 14–20 10.1645/0022-3395(2003)089[0014:TSGEAP]2.0.CO;2 12659297

[B95] Kubeš M. Fuchsberger N. Labuda M. Žuffová E. Nuttall P. A. (1994). Salivary gland extracts of partially fed *Dermacentor reticulatus* ticks decrease natural killer cell activity *in vitro*. Immunology 82, 113–116 8045588 PMC1414839

[B96] Kung F. Anguita J. Pal U. (2013). *Borrelia burgdorferi* and tick proteins supporting pathogen persistence in the vector. Future Microbiol. 8, 41–56 10.2217/fmb.12.121 23252492 PMC3564579

[B97] Kuthejlová M. Kopecký J. Štìpánová G. Macela A. (2001). Tick salivary gland extract inhibits killing of *Borrelia afzelii* spirochaetes by mouse macrophages. Infect. Immun. 69, 575–578 10.1128/IAI.69.1.575-578.2001 11119556 PMC97922

[B98] Labuda M. Austyn J. M. Zuffova E. Kozuch O. Fuchsberger N. Lysy J. (1996). Importrance of localized skin infection in tick-borne encephalitis virus transmission. Virology 219, 357–366 10.1006/viro.1996.0261 8638401

[B99] Labuda M. Jones L. D. Williams T. Nuttall P. A. (1993a). Enhancement of tick-borne encephalitis virus transmission by tick salivary gland extracts. Med. Vet. Entomol. 7, 193–196 10.1111/j.1365-2915.1993.tb00674.x 8481537

[B100] Labuda M. Nuttall P. A. Kozuch O. Eleckova E. Williams T. Zuffova E. (1993b). Nonviraemic transmission of tick-borne encephalitis virus: a mechanism for arbovirus survival in nature. Experientia 49, 802–805 10.1007/BF01923553 8405306

[B101] Lawrie C. H. Randolph S. E. Nuttall P. A. (1999). *Ixodes* ticks: serum species sensitivity of anti-complement activity. Exp. Parasitol. 93, 207–214 10.1006/expr.1999.4456 10600446

[B102] Leboulle G. Crippa M. Decrem Y. Mejri N. Brossard M. Bollen A. (2002). Characterization of a novel salivary immunosuppressive protein from *Ixodes ricinus* ticks. J. Biol. Chem. 277, 10083–10089 10.1074/jbc.M111391200 11792703

[B103] Lieskovská J. Kopecký J. (2012a). Tick saliva suppresses IFN signalling in dendritic cells upon *Borrelia afzelii* infection. Parasite Immunol. 34, 32–39 10.1111/j.1365-3024.2011.01345.x 22097894

[B104] Lieskovská J. Kopecký J. (2012b). Effect of tick saliva on signalling pathways activated by TLR-2 ligand and *Borrelia afzelii* in dendritic cells. Parasite Immunol. 34, 421–429 10.1111/j.1365-3024.2012.01375.x 22709526

[B105] Limo M. K. Voigt W. P. Tumbo-Oeri A. Njogu R. M. Ole-Moi Yoi O. N. (1991). Purification and characterization of an anticoagulant from the salivary glands of the ixodid tick *Rhipicephalus appendiculatus*. Exp. Parasitol. 72, 418–429 10.1016/0014-4894(91)90088-E 2026216

[B106] Liyou N. Hamilton S. Elvin C. Willadsen P. (1999). Clonng, expression of ecto 5-nucleotidase from cattle tick *Boophilus microplus*. Insect Mol. Biol. 8, 257–266 10.1046/j.1365-2583.1999.820257.x 10380109

[B107] Macedo-Ribeiro S. Almeida C. Calisto B. M. Friedrich T. Mentele R. Stürzebecher J. (2008). Isolation, cloning and structural characterisation of boophilin, a multifunctional Kunitz-type proteinase inhibitor from the cattle tick. PLoS ONE 3:e1624 10.1371/journal.pone.0001624 18286181 PMC2230226

[B108] Macháčková M. Oborník M. Kopecký J. (2006). Effect of salivary gland extract from *Ixodes ricinus* ticks on the proliferation of *Borrelia burgdorferi* sensu stricto *in vivo*. Folia Parasitol. 53, 153–158 16898130

[B109] Mans B. J. (2010). Evolution of vertebrate hemostatic and inflammatory control mechanisms in blood-feeding arthropods. J. Innate Immun. 10.1159/000321599 20980728

[B110] Mans B. J. Andersen J. F. Francischetti I. M. B. Valenzuela J. G. Schwan T. G. Pham V. M. (2008). Comparative sialomics between hard and soft ticks: implications for the evolution of blood-feeding behavior. Insect Biochem. Mol. Biol. 38, 42–58 10.1016/j.ibmb.2007.09.003 18070664 PMC2211429

[B111] Mans B. J. Coetzee J. Louw A. I. Gaspar A. R. M. Neitz A. W. H. (2000). Disaggregation of aggregated platelets by apyrase from the tick *Ornithodoros savignyi* (Acari: Argasidae). Exp. Appl. Acarol. 24, 271–282 10.1023/A:1006440714276 11110238

[B112] Mans B. J. Louw A. I. Gaspar A. R. M. D. Neitz A. W. H. (1998a). Apyrase activity and platelet aggregation inhibitors in the tick *Ornithodoros savignyi*. Exp. Appl. Acarol. 22, 353–366 10.1023/A:1024517209621 9652096

[B113] Mans B. J. Louw A. I. Gaspar A. R. M. D. Neitz A. W. H. (1998b). Purification and characterisation of apyrase from the tick, *Ornithodoros savignyi*. Comp. Biochem. Physiol. B Biochem. Mol. Biol. 120, 617–624 10.1016/S0305-0491(98)10061-5 14598857

[B114] Mans B. J. Louw A. I. Neitz A. W. H. (2002a). Evolution of hematophagy in ticks: common origins for blood coagulation and platelet aggregation inhibitors from soft ticks of the genus *Ornithodoros*. Mol. Biol. Evol. 19, 1695–1705 10.1093/oxfordjournals.molbev.a003992 12270896

[B115] Mans B. J. Louw A. I. Neitz A. W. H. (2002b). Savignygrin, a platelet aggregation inhibitor from the soft tick *Ornithodoros savignyi*, present the RGD integrin recognition motif on the Kunitz-BPTI fold. J. Biol. Chem. 277, 21371–21378 10.1074/jbc.M112060200 11932256

[B116] Mans B. J. Louw A. I. Neitz A. W. H. (2002c). Disaggregation of aggregated platelets by savignygrin, a alphaIIbeta3 antagonist from *Ornithodoros savignyi*. Exp. Appl. Acarol. 27, 231–239 10.1023/A:1021613001297 12593588

[B117] Mans B. J. Neitz A. W. H. (2004). Adaptation of ticks to a blood-feeding environment: evolution from a functional perspective. Insect Biochem. Molec. Biol. 34, 1–17 10.1016/j.ibmb.2003.09.002 14723893

[B118] Marchal C. Luft B. J. Yang X. Sibilia J. Jaulhac B. Boulanger N. (2009). Defensin is suppressed by tick salivary gland extract during the *in vitro* interaction of resident skin cells with *Borrelia burgdorferi*. J. Invest. Dermatol. 129, 2515–2517 10.1038/jid.2009.73 19340008

[B119] Marchal C. Schramm F. Kern A. Luft B. J. Yang X. Schuijt T. (2011). Antialarmin effect of tick saliva during the transmission of *Lyme* disease. Infect. Immun. 79, 774–785 10.1128/IAI.00482-10 21134970 PMC3028856

[B120] Maritz-Olivier C. Stutzer C. Jongejan F. Neitz A. W. Gaspar A. R. (2007). Tick anti-hemostatics: targets for future vaccines and therapeutics. Trends Parasitol. 23, 397–407 10.1016/j.pt.2007.07.005 17656153

[B121] Mejri N. Franscini N. Rutti B. Brossard M. (2001). Th2 polarization of the immune response of Balb/c mice to *Ixodes ricinus* instars, importance of several antigens in activation of specific Th2 subpopulations. Parasite Immunol. 23, 61–69 10.1046/j.1365-3024.2001.00356.x 11240897

[B122] Mejri N. Rutti B. Brossard M. (2002). Immunosuppressive effects of *Ixodes ricinus* tick saliva or salivary gland extracts on innate and acquired immune response of BALB/c mice. Parasitol. Res. 88, 192–197 10.1007/s00436-001-0515-1 11954903

[B123] Montgomery R. R. Lusitani D. de Boisfleury Chevance A. Malawista S. E. (2004). Tick saliva reduces adherence and area of human neutrophils. Infect. Immun. 72, 2989–2994 10.1128/IAI.72.5.2989-2994.2004 15102811 PMC387908

[B124] Mori A. Konnai S. Yamada S. Hidano A. Murase Y. Ito T. (2010). Two novel Salp15-like immunosuppressant genes from salivary glands of *Ixodes persulcatus* Schulze tick. Insect Mol. Biol. 19, 359–365 10.1111/j.1365-2583.2010.00994.x 20201978

[B125] Motoyashiki T. Tu A. T. Ayimov D. A. Ibragim K. (2003). Isolation of anticoagulant from the venom of tick, *Boophilus calcaratus*, from Uzbekistan. Thromb. Res. 110, 235–241 10.1016/S0049-3848(03)00409-2 14512088

[B126] Mulenga A. Sugino M. Nakajima M. Sugimoto C. Onuma M. (2001). Tick-encoded serine proteinase inhibitors (serpins); potential target antigens for tick vaccine development. J. Vet. Med. Sci. 63, 1063–1069 10.1292/jvms.63.1063 11714020

[B127] Mulenga A. Tsuda A. Onuma M. Sugimoto C. (2003). Four serine proteinase inhibitors (serpin) from the brown ear tick, *Rhipicephalus appendiculatus*; cDNA cloning and preliminary characterisation. Insect Biochem. Mol. Biol. 33, 267–276 10.1016/S0965-1748(02)00240-0 12535684

[B128] Narasimhan S. DePonte K. Marcantoni N. Liang X. Royce T. E. Nelson K. F. (2007a). Immunity against *Ixodes scapularis* salivary proteins expressed within 24 hours of attachment thwarts tick feeding and impairs *Borrelia* transmission. PLoS ONE 2:e451 10.1371/journal.pone.0000451 17505544 PMC1866177

[B129] Narasimhan S. Sukumaran B. Bozdogan U. Thomas V. Liang X. DePonte K. (2007b). A tick antioxidant facilitates the *Lyme* disease agent's successful migration from the mammalian host to the arthropod vector. Cell Host Microbe 2, 7–18 10.1016/j.chom.2007.06.001 18005713 PMC2699493

[B130] Narasimhan S. Koski R. A. Beaulieu B. Anderson J. F. Ramamoorthi N. Kantor F. (2002). A novel family of anticoagulants from the saliva of *Ixodes scapularis*. Insect Mol. Biol. 11, 641–650 10.1046/j.1365-2583.2002.00375.x 12421422

[B131] Narasimhan S. Montgomery R. R. DePonte K. Tschudi C. Marcantonio N. Anderson J. F. (2004). Disruption of *Ixodes scapularis* anticoagulation by using RNA interference. Proc. Natl. Acad. Sci. U.S.A. 101, 1141–1146 10.1073/pnas.0307669100 14745044 PMC337020

[B132] Nazario S. Das S. De Silva A. M. DePonte K. Marcantonio N. Anderson J. F. (1998). Prevention of *Borrelia burgdorferi* transmission in guinea pigs by tick immunity. Am. J. Trop. Med. Hyg. 58, 780–785 9660463 10.4269/ajtmh.1998.58.780

[B133] Nienaber J. Gaspar A. R. M. Neitz A. W. H. (1999). Savignin, a potent thrombin inhibitor isolated from the salivary glands of the tick *Ornithodoros savignyi* (*Acari: Argasidae*). Exp. Parasitol. 93, 82–91 10.1006/expr.1999.4448 10502470

[B134] Nunn M. A. Sharma A. Paesen G. C. Adamson S. Lissina O. Willis A. C. (2005). Complement inhibitor of C5 activation from the soft tick *Ornithodoros moubata*. J. Immunol. 174, 2084–2091 15699138 10.4049/jimmunol.174.4.2084

[B135] Nuttall P. A. Labuda M. (2004). Tick-host interactions: saliva-activated transmission. Parasitology 129, S177–S189 10.1017/S0031182004005633 15938511

[B136] Nuttall P. A. Labuda M. (2008). Saliva-assisted transmission of tick-borne pathogens, in Ticks: Biology, Disease and Control, eds Bowman A. S. Nuttall P. A. (Cambridge; New York, NY: Cambridge University Press), 205–219 10.1017/CBO9780511551802.011

[B137] Ogden N. H. Nuttall P. A. Randolph S. E. (1997). Natural Lyme disease cycle maintained via sheep by co-feeding ticks. Parasitology 115, 591–599 10.1017/S0031182097001868 9488870

[B138] Oliveira C. J. Sa-Nunes A. Francischetti I. M. Carregaro V. Anatriello E. Silva J. S. (2011). Deconstructing tick saliva: non-protein molecules with potent immunomodulatory properties. J. Biol. Chem. 286, 10960–10969 10.1074/jbc.M110.205047 21270122 PMC3064151

[B139] Paesen G. Adams P. L. Harlos K. Nuttall P. A. Stuart D. I. (1999). Tick histamine-binding proteins: isolation, cloning, and threedimensional structure. Mol. Cell 3, 661–671 10.1016/S1097-2765(00)80359-7 10360182

[B140] Paesen G. C. Siebold S. Harlos K. Peacey M. F. Nuttall P. A. Stuart D. I. (2007). A tick protein with a modified Kunitz fold inhibits human tryptase. J. Mol. Biol. 368, 1172–1186 10.1016/j.jmb.2007.03.011 17391695

[B141] Pal U. Li X. Wang T. Montgomery R. R. Ramamoorthi N. Desilva A. M. (2004). TROSPA, an *Ixodes scapularis* receptor for *Borrelia burgdorferi*. Cell 119, 457–468 10.1016/j.cell.2004.10.027 15537536

[B142] Pechová J. Štépánová G. Kovář L. Kopecký J. (2002). Tick salivary gland extract-activated transmission of *Borrelia afzelii* spirochaetes. Folia Parasitol. 49, 153–159 12194488

[B143] Piesman J. Happ C. M. (2001). The efficacy of co-feeding as a means of maintaining *Borrelia burgdorferi*: a North American model system. J. Vector Ecol. 26, 216–220 11813659

[B144] Preston S. G. Majtán J. Kouremenou C. Rysnik O. Burger L. F. Cabezas Cruz A. (2013). Novel immunomodulators from hard ticks selectively reprogramme human dendritic cell responses. PLoS Pathog. 9:e1003450 10.1371/journal.ppat.1003450 23825947 PMC3695081

[B145] Prevot P. P. Adam B. Boudjeltia K. Z. Brossard M. Lins L. Cauchie P. (2006). Anti-hemostatic effects of a serpin from the saliva of the tick *Ixodes ricinus*. J. Biol. Chem. 281, 26361–26369 10.1074/jbc.M604197200 16672226

[B146] Radolf J. D. Caimano M. J. Stevenson B. Hu L. T. (2012). Of ticks, mice and men: understanding the dual-host lifestyle of Lyme disease spirochaetes. Nat. Rev. Microbiol. 10, 87–99 22230951 10.1038/nrmicro2714 PMC3313462

[B147] Ramachandra R. N. Wikel S. K. (1992). Modulation of host-immune responses by ticks *Acari: Ixodidae*: effects of salivary gland extracts on host macrophages and *lymphocyte* cytokine production. J. Med. Entomol. 5, 818–826 1404261 10.1093/jmedent/29.5.818

[B148] Ramamoorthi N. Narasimhan S. Pal U. Bao F. Yang X. F. Fish D. (2005). The *Lyme* disease agent exploits a tick protein to infect the mammalian host. Nature 436, 573–577 10.1038/nature03812 16049492 PMC4306560

[B149] Randolph S. E. (2009). Tick-borne disease systems emerge from the shadows: the beauty lies in molecular detail, the message in epidemiology. Parasitology 136, 1403–1413 10.1017/S0031182009005782 19366480

[B150] Randolph S. E. (2011). Transmission of tick-borne pathogens between co-feeding ticks: milan Labuda's enduring paradigm. Ticks Tick Borne Dis. 2, 179–182 10.1016/j.ttbdis.2011.07.004 22108009

[B151] Randolph S. E. Gern L. Nuttall P. A. (1996). Co-feeding ticks: epidemiological significance for tick-borne pathogen transmission. Parasitol. Today 12, 472–479 10.1016/S0169-4758(96)10072-7 15275266

[B152] Randolph S. E. Miklisova D. Lysy J. Rogers D. J. Labuda M. (1999). Incidence from coincidence: patterns of tick infestations on rodents facilitate transmission of tick-borne encephalitis virus. Parasitology 118, 177–186 10.1017/S0031182098003643 10028532

[B153] Ribeiro J. M. Alarcon-Chaidez F. Francischetti I. M. Mans B. J. Mather T. N. Valenzuela J. G. (2006). An annotated catalog of salivary gland transcripts from *Ixodes scapularis* ticks. Insect Biochem. Mol. Biol. 36, 111–129 10.1016/j.ibmb.2005.11.005 16431279

[B154] Ribeiro J. M. Evans P. M. McSwain J. L. Sauer J. R. (1992). *Amblyomma americanum*: characterization of salivary prostaglandins E_2_ and F_2*alpha*_ by RP-HPLC/bioassay and gas chromatography-mass spectrometry. Exp. Parasitol. 74, 112–116 10.1016/0014-4894(92)90145-Z 1730268

[B155] Ribeiro J. M. Francischetti I. M. (2003). Role of arthropod saliva in blood feeding: sialome and post-sialome perspectives. Annu. Rev. Entomol. 48, 73–88 10.1146/annurev.ento.48.060402.102812 12194906

[B156] Ribeiro J. M. C. (1995). How ticks make a living. Parasitol. Today 11, 91–93 10.1016/0169-4758(95)80162-6 15275359

[B157] Ribeiro J. M. C. Endris T. M. Endris R. (1991). Saliva of the soft tick, *Ornithodoros moubata*, contains anti-platelet and apyrase activities. Comp. Biochem. Physiol. A 100, 109–112 10.1016/0300-9629(91)90190-N 1682082

[B158] Ribeiro J. M. C. Mather T. N. (1998). *Ixodes scapularis*: salivary kininase activity is a metallo dipeptidyl carboxypeptidase. Exp. Parasitol. 89, 213–221 10.1006/expr.1998.4296 9635445

[B159] Ribeiro J. M. C. Makoul G. T. Robinson D. R. (1988). *Ixodes dammini:* evidence for salivary prostacyclin secretion. J. Parasitol. 74, 1068–1069 10.2307/3282240 3057165

[B160] Ribeiro J. M. C. Makoul G. T. Robinson D. R. Spielman A. (1985). Antihaemostatic, antiinflammatory and immunosuppressive properties of the saliva of a tick, *Ixodes dammini*. J. Exp. Med. 161, 332–344 10.1084/jem.161.2.332 2982989 PMC2187567

[B161] Ribeiro J. M. C. Weis J. J. Telford S. R. (1990). Saliva of the tick *Ixodes dammini* inhibits neutrophil functions. Exp. Parasitol. 70, 382–388 10.1016/0014-4894(90)90121-R 2157607

[B162] Richter D. Allgöwer R. Matuschka F. R. (2002). Co-feeding transmission and its contribution to the perpetuation of the *Lyme* disease spirochete *Borrelia afzelii*. Emerg. Infect. Dis. 8, 1421–1425 10.3201/eid0812.010519 12498658 PMC2738522

[B163] Sá-Nunes A. Bafica A. Antonelli L. R. Young Choi E. Francischetti I. M. B. Andersen J. F. (2009). The immunomodulatory action of sialostatin L on dendritic cells reveals its potential to interfere with autoimmunity. J. Immunol. 182, 7422–7429 10.4049/jimmunol.0900075 19494265 PMC2694955

[B164] Sangamnatdej S. Paesen G. C. Slovak M. Nuttall P. A. (2002). A high affinity serotonin- and histamine-binding lipocalin from tick saliva. Insect Mol. Biol. 11, 79–86 10.1046/j.0962-1075.2001.00311.x 11841505

[B165] Sant Anna Azzolini S. Sasaki S. D. Torquato R. J. Andreotti R. Andreotti E. Tanaka A. S. (2003). *Rhipicephalus sanguineus* trypsin inhibitors present in the tick larvae: isolation, characterization, and partial primary structure determination. Arch. Biochem. Biophys. 417, 176–182 10.1016/S0003-9861(03)00344-8 12941299

[B166] Sauer J. R. Essenberg R. C. Bowman A. S. (2000). Salivary glands in ixodid ticks: control and mechanism of secretion. J. Insect Physiol. 46, 1069–1078 10.1016/S0022-1910(99)00210-3 10817833

[B167] Sauer J. R. McSwain J. L. Bowman A. S. Essenberg R. C. (1995). Tick salivary gland physiology. Annu. Rev. Entomol. 40, 245–267 10.1146/annurev.en.40.010195.001333 7810988

[B168] Schoeler G. B. Bergman D. K. Manweiler S. K. Wikel S. K. (2000a). Influence of soluble proteins from the salivary glands of ixodid ticks on *in-vitro* proliferative responses of *lymphocytes* from BALB/c and C3H/HeN mice. Ann. Trop. Med. Parasitol. 94, 507–518 10983564 10.1080/00034983.2000.11813570

[B169] Schoeler G. B. Manweiler S. A. Wikel S. K. (2000b). Cytokine responses of C3H/HeN mice infested with *Ixodes scapularis* or *Ixodes pacificus* nymphs. Parasite Immunol. 22, 39–48 10.1046/j.1365-3024.2000.00272.x 10607289

[B170] Schoeler G. B. Manweiler S. A. Wikel S. K. (1999). *Ixodes scapularis*: effects of repeated infestations with pathogen-free nymphs on macrophage and T lymphocyte cytokine responses of BALB/c and C3H/HeN mice. Exp. Parasitol. 92, 239–248 10.1006/expr.1999.4426 10425152

[B171] Schoeler G. B. Wikel S. K. (2001). Modulation of host immunity by haematophagous arthropods. Ann. Trop. Med. Parasitol. 95, 755–771 10.1080/0003498012011118 11784430

[B172] Schuijt T. J. Bakhtiari K. Daffre S. Deponte K. Wielders S. J. Marquart J. A. (2013). Factor Xa activation of factor V is of paramount importance in initiating the coagulation system: lessons from a tick salivary protein. Circulation 128, 254–266 10.1161/CIRCULATIONAHA.113.003191 23817575 PMC3826089

[B173] Schuijt T. J. Coumou J. Narasimhan S. Dai J. Deponte K. Wouters D. (2011a). A tick mannose-binding lectin inhibitor interferes with the vertebrate complement cascade to enhance transmission of the *Lyme* disease agent. Cell Host Microbe 10, 136–146 10.1016/j.chom.2011.06.010 21843870 PMC3170916

[B174a] Schuijt T. J. Narasimhan S. Daffre S. DePonte K. Hovius J. W. R. van't Veer C. (2011b). Identification and characterization of *Ixodes scapularis* antigens that elicit tick immunity using yeast surface display. PLoS ONE 6:e15926 10.1371/journal.pone.0015926 21246036 PMC3016337

[B174] Severinová J. Salát J. Krocová Z. Reznícková J. Demová H. Horká H. (2005). Co-inoculation of *Borrelia afzelii* with tick salivary gland extract influences distribution of immunocompetent cells in the skin and lymph nodes of mice. Folia Microbiol. 50, 457–463 10.1007/BF02931430 16475508

[B175] Skallová A. Iezzi G. Ampenberger F. Kopf M. Kopecký J. (2008). Tick saliva inhibits dendritic cell migration, maturation, and function while promoting development of Th2 responses. J. Immunol. 180, 6186–6192 18424740 10.4049/jimmunol.180.9.6186

[B176] Slámová M. Skallová A. Páleníková J. Kopecký J. (2011). Effect of tick saliva on immune interactions between *Borrelia afzelii* and murine dendritic cells. Parasite Immunol. 33, 654–660 10.1111/j.1365-3024.2011.01332.x 21910742

[B177] Sonenshine D. E. (1991). Biology of Ticks. Vol. 1, New York, Oxford: Oxford University Press

[B178] Steen N. A. Barker S. C. Alewood P. F. (2005). Proteins in the saliva of the *Ixodida (ticks)*: pharmacological features and biological significance. Toxicon 47, 1–20 10.1016/j.toxicon.2005.09.010 16364387

[B179] Sukumaran B. Narasimhan S. Anderson J. F. DePonte K. Marcantonio N. Krishnan M. N. (2006). An *Ixodes scapularis* protein required for survival of *Anaplasma phagocytophilum* in tick salivary glands. J. Exp. Med. 203, 1507–1517 10.1084/jem.20060208 16717118 PMC2118316

[B180] Tanaka A. S. Andreotti R. Gomes A. Torquato R. J. S. Sampaio M. U. Sampaio C. A. M. (1999). A Double-headed serine protease inhibitor—human plasma kallikrein and elastase inhibitor—from *Boophilus microplus larvae*. Immunopharmacology 45, 171–177 10.1016/S0162-3109(99)00074-0 10615008

[B181] Titus R. G. Bishop J. V. Mejia J. S. (2006). The immunomodulatory factors of arthropod saliva and the potential for these factors to serve as vaccine targets to prevent pathogen transmission. Parasite Immunol. 28, 131–141 16542315 10.1111/j.1365-3024.2006.00807.x

[B183] Tyson K. Elkins C. Patterson H. Fikrig E. de Silva A. (2007). Biochemical and functional characterization of Salp20, an *Ixodes scapularis* tick salivary protein that inhibits the complement pathway. Insect Mol. Biol. 16, 469–479 10.1111/j.1365-2583.2007.00742.x 17651236

[B184] Valenzuela J. G. (2002). High-throughput approaches to study salivary proteins and genes from vectors of disease. Insect Biochem. Mol. Biol. 32, 1199–1209 10.1016/S0965-1748(02)00083-8 12225911

[B185] Valenzuela J. G. (2004). Exploring tick saliva: from biochemistry to “sialomes” and functional genomics. Parasitology 129, S83–S94 10.1017/S0031182004005189 15938506

[B186] Valenzuela J. G. Charlab R. Mather T. N. Ribeiro J. M. (2000). Purification, cloning, and expression of a novel salivary anticomplement protein from the tick, *Ixodes scapularis*. J. Biol. Chem. 275, 18717–18723 10.1074/jbc.M001486200 10749868

[B187] Valenzuela J. G. Francischetti I. M. B. Pham V. M. Garfield M. K. Mather T. N. Ribeiro J. M. C. (2002). Exploring the sialome of the tick *Ixodes scapularis*. J. Exp. Biol. 205, 2843–2864 12177149 10.1242/jeb.205.18.2843

[B188] Vančová I. Hajnická V. Slovák M. Kocáková P. Paesen G. C. Nutall P. A. (2010a). Evasin-3-like anti-chemokine activity in salivary gland extracts of ixodid ticks during blood-feeding: a new target for tick control. Parasite Immunol. 32, 460–463 10.1111/j.1365-3024.2010.01203.x 20500677

[B189] Vančová I. Hajnická V. Slovák M. Nutall P. A. (2010b). Anti-chemokine activities of ixodid ticks depend on tick species, developmental stage, and duration of feeding. Vet. Parasitol. 167, 274–278 10.1016/j.vetpar.2009.09.029 19836889

[B190] Van de Locht A. Stubbs M. T. Bode W. Friedrich T. Bollschweiler C. Hoffken W. (1996). The ornithodorin-thrombin crystal structure a key to the TAP engima. EMBO J. 15, 6011–6017 8947023 PMC452422

[B191] Wang H. Nuttall P. A. (1999). Immunoglobulin-binding proteins in ticks: new target for vaccine development against a blood-feeding parasite. Cell. Mol. Life Sci. 56, 286–295 10.1007/s000180050430 11212356 PMC11147071

[B192] Wang X. Coons L. B. Taylor D. B. Stevens S. E. Gartner T. K. (1996). Variabilin, a novel RGD-containing antagonist of glycoprotein IIb-IIIa and platelet aggregation inhibitor from the hard tick *Dermacentor variabilis*. J. Biol. Chem. 271, 17785–17790 10.1074/jbc.271.30.17785 8663513

[B193] Waxman L. Connolly T. M. (1993). Isolation of an inhibitor selective for collagen-stimulated platelet aggregation from the soft tick *Ornithodoros moubata*. J. Biol. Chem. 268, 5445–5449 8449906

[B194] Waxman L. Smith D. E. Arcuri K. E. Vlasuk G. P. (1990). Tick Anticoagulant Peptide (TAP) is a novel inhibitor of blood coagulation factor Xa. Science 248, 593–596 10.1126/science.2333510 2333510

[B195] Wikel S. K. (1996). The Immunology of Host-Ectoparasitic Arthropod Relationships. Oxon: CAB International

[B196] Wikel S. K. (1999). Modulation of the host immune system by ectoparasitic arthropods. Bioscience 49, 311–320 10.2307/1313614

[B197] Wikel S. K. Alarcon-Chaidez F. J. (2001). Progress toward molecular characterization of ectoparasite modulation of host immunity. Vet. Parasitol. 101, 275–287 10.1016/S0304-4017(01)00556-8 11707302

[B198] Wikel S. K. Ramachandra R. N. Bergman D. K. Burkot T. R. Piesman J. (1997). Infestation with pathogen-free nymphs of the tick *Ixodes scapularis* induces host resistance to transmission of *Borrelia burgdorferi* by ticks. Infect. Immun. 65, 335–338 8975935 10.1128/iai.65.1.335-338.1997 PMC174599

[B199] Willadsen P. (2004). Anti-tick vaccines. Parasitology 129, S367–S387 10.1017/S0031182003004657 15938519

[B200] Wu J. Wang Y. Liu H. Yang H. Ma D. Li J. (2010). Two immunoregulatory peptides with antioxidant activity from tick salivary glands. J. Biol. Chem. 285, 16606–16613 10.1074/jbc.M109.094615 20178988 PMC2878058

[B201] Zeidner N. Dreitz M. Belasco D. Fish D. (1996). Suppression of acute *Ixodes scapularis*-induced *Borrelia burgdorferi* infection using tumor necrosis factor-alpha, interleukin-2, and interferon-gamma. J. Infect. Dis. 173, 187–195 10.1093/infdis/173.1.187 8537658

[B202] Zeidner N. Lamine Mbow M. Dolan M. Massung R. Baca E. Piesman J. (1997). Effects of *Ixodes scapularis* and *Borrelia burgdorferi* on modulation of the host immune response: induction of a TH2 cytokine response in *Lyme* disease-susceptible (C3H/HeJ) mice but not in disease-resistant (BALB/c) mice. Infect. Immun. 65, 3100–3106 9234760 10.1128/iai.65.8.3100-3106.1997 PMC175437

[B203] Zeidner N. S. Schneider B. S. Nuncio M. S. Gern L. Piesman J. (2002). Coinoculation of *Borrelia* spp. with tick salivary gland lysate enhances spirochaete load in mice and is tick species-specific. J. Parasitol. 88, 1276–1278 12537131 10.1645/0022-3395(2002)088[1276:COBSWT]2.0.CO;2

[B204] Yu D. Liang J. Yu H. Wu H. Xu C. Liu J. (2006). A tick B-cell inhibitory protein from salivary glands of the hard tick, *Hyalomma asiaticum asiaticum*. Biochem. Biophys. Res. Commun. 343, 585–590 10.1016/j.bbrc.2006.02.188 16554026

